# ZFP541 and KCTD19 regulate chromatin organization and transcription programs for male meiotic progression

**DOI:** 10.1111/cpr.13567

**Published:** 2023-11-03

**Authors:** Xu Zhou, Kailun Fang, Yanlei Liu, Weidong Li, Yingjin Tan, Jiaming Zhang, Xiaoxia Yu, Guoqiang Wang, Yanan Zhang, Yongliang Shang, Liangran Zhang, Charlie Degui Chen, Shunxin Wang

**Affiliations:** ^1^ Advanced Medical Research Institute Shandong University Jinan Shandong China; ^2^ State Key Laboratory of Molecular Biology, Shanghai Key Laboratory of Molecular Andrology, CAS Center for Excellence in Molecular Cell Science Shanghai Institute of Biochemistry and Cell Biology, Chinese Academy of Sciences Shanghai China; ^3^ State Key Laboratory of Reproductive Medicine and Offspring Health, Center for Reproductive Medicine Shandong University Jinan Shandong China; ^4^ Center for Cell Structure and Function, Shandong Provincial Key Laboratory of Animal Resistance Biology, College of Life Sciences Shandong Normal University Jinan Shandong China; ^5^ Key Laboratory of Reproductive Endocrinology of Ministry of Education, National Research Center for Assisted Reproductive Technology and Reproductive Genetics Shandong University Jinan Shandong China; ^6^ Shandong Key Laboratory of Reproductive Medicine, Shandong Provincial Clinical Research Center for Reproductive Health Shandong Technology Innovation Center for Reproductive Health Jinan Shandong China

## Abstract

The successful progression of meiosis prophase I requires integrating information from the structural and molecular levels. In this study, we show that ZFP541 and KCTD19 work in the same genetic pathway to regulate the progression of male meiosis and thus fertility. The *Zfp541* and/or *Kctd19* knockout male mice show various structural and recombination defects including detached chromosome ends, aberrant localization of chromosome axis components and recombination proteins, and globally altered histone modifications. Further analyses on RNA‐seq, ChIP‐seq, and ATAC‐seq data provide molecular evidence for the above defects and reveal that ZFP541/KCTD19 activates the expression of many genes by repressing several major transcription repressors. More importantly, we reveal an unexpected role of ZFP541/KCTD19 in directly modulating chromatin organization. These results suggest that ZFP541/KCTD19 simultaneously regulates the transcription cascade and chromatin organization to ensure the coordinated progression of multiple events at chromosome structural and biochemical levels during meiosis prophase I.

## Graphical abstract

The role of the ZFP541‐KCTD19 complex in spermatogenesis is unclear. Zhou et al. reveal that ZFP541‐KCTD19 suppresses several major transcription repressors to maintain the expression of many genes required for meiosis. Meanwhile, ZFP541‐KCTD19 can also directly modulate chromatin organization and increase the levels of histone acetylation and ubiquitination. Therefore, ZFP541‐KCTD19 integrates information from DNA molecular and chromosome structural levels for the coordinated progression of multiple events during spermatogenesis.

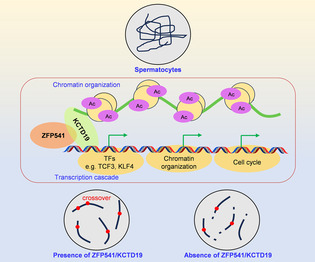

## 1 INTRODUCTION

Meiosis is a specialized cell division through which diploid progenitor cells generate haploid gametes. During meiosis, DNA is replicated once, followed by two successive rounds of chromosome segregation. Homologous chromosomes (homologs) segregate at meiosis I and sister chromatids (sisters) segregate at meiosis II. Crossovers (COs) formed between homologs are required to ensure faithful chromosome segregation and facilitate genetic diversity.^1^, ^2^

Meiosis is characterized by an extended prophase I with two unique events: homolog synapsis and CO recombination.^1^, ^2^, ^3^, ^4^ Each step of meiotic recombination, from the occurrence of DNA double‐strand breaks (DSBs) to the formation of crossovers, is integrated into and tightly regulated by meiotic chromosome architecture, which is organized as an array of loops on the base of the chromosome axis.^5^, ^6^, ^7^, ^8^, ^9^ Accompanied by meiotic recombination, chromosomes undergo dynamic changes during prophase I.^1^, ^2^, ^4^ At leptotene, along with axes developing, pre‐DSB complexes in chromatin loops are recruited and tethered to axes for DSB formation. Usually, a great number of programmed DSBs are generated in each nucleus.^10^, ^11^, ^12^, ^13^ After end resection, DMC1 and RAD51 are recruited to resected single‐stranded DNAs (ssDNAs), promoting ssDNA to invade homologous chromosomes to form D‐loops.^14^ The synaptonemal complex (SC) is initiated between homologs probably at a subset of these recombination intermediates at zygotene. When SC assembles on whole chromosome axes, that is, synapsis is complete, cells enter pachytene. Only a minority of D‐loops are selected to be CO sites and visualized as MLH1 foci at pachytene. The rest develops into noncrossovers (NCOs).^1^, ^15^, ^16^, ^17^, ^18^ Upon disassembly of SC, cells enter diplotene and homologs are physically linked by chiasmata, the cytological manifestation of COs. After that, chromosomes are further condensed and aligned on the equatorial plates at metaphase I (MI). The tight association between recombination, SC, and chromosome organization, is required for the proper progression of meiosis prophase.^5^, ^7^, ^9^

Many studies show that dramatic changes in gene expression programs occur on highly compacted chromosomes and are required for the proper progression of meiotic prophase.^19^, ^20^, ^21^, ^22^, ^23^ Genes required for post‐meiotic stages are transcribed during pachytene.^24^, ^25^, ^26^ Several reports indicate that ZFP541 (zinc finger protein 541) works with KCTD19 (potassium channel tetramerization domain containing 19) to play an important role in regulating transcriptional programs for the progression of male meiosis.^27^, ^28^, ^29^, ^30^, ^31^, ^32^ ZFP541 contains five C2H2 zinc finger motifs and an ELM2‐SANT domain that binds to DNA and interacts with HDAC1/2, respectively.^27^ The N‐terminus of KCTD19 contains two BTB/POZ domains, which usually mediate protein–protein interaction and functions in many processes including transcription repression, tetramerization and gating of ion channels, and interaction with cullin E3 ubiquitin ligase complex.^33^ These studies also suggest that KCTD19 probably works downstream of ZFP541 based on several pieces of evidence^28^, ^29^, ^30^, ^31^: (1) *Kctd19* ^*−/−*^ spermatocytes show fewer defects and arrested at a later stage than those of *Zfp541* ^*−/−*^, (2) ZFP541 is expressed probably as early as leptotene/zygotene, however, KCTD19 is expressed only after early pachytene in spermatocytes. These studies also raise interesting issues and arguments that are remained unresolved. (1) ZFP541 can interact with KCTD19 and they show functional differences. However, it is unclear whether they have functional redundancy and whether they work in the same genetic pathway to regulate meiosis. (2) Transcriptome analysis shows ZFP541 can directly bind to promoters of a small number of genes and repress their expression. However, how the expression of a much larger number of genes is regulated by ZFP541 is unknown. (3) It is unknown whether and how they are involved in the meiotic recombination process.

It is widely accepted that a loop extrusion process organizes chromatin. In this process, anchors block the extrusion process and largely determine the size of loops.^34^, ^35^ Transcription factors can work through the transcription activity/process to adjust the locations of anchor proteins and thus modulate chromatin organization. Some transcription factors can also work as anchor proteins to directly modulate chromatin organization.^36^, ^37^, ^38^ It would be interesting to know whether ZFP541/KCTD19 can directly regulate chromatin organization in addition to its role as a transcription factor.

Here, we systematically investigated the roles of ZFP541 and KCTD19 in male meiosis using the two single and one double‐knockout mice in parallel. We showed that ZFP541 and KCTD19 work in the same genetic pathway and are required for the completion of male meiosis and thus male fertility. The knockout mice showed various structural and recombination defects including detached chromosome ends, aberrant localization of chromosome axis components, and histone modifications. Analyses on RNA‐seq, ChIP‐seq, and ATAC‐seq not only confirmed those defects but also revealed their roles in regulating transcription cascade and chromatin organization. These results support the proposal that ZFP541/KCTD19 simultaneously regulates chromatin organization and transcription program to coordinate meiosis progression at structural and molecular levels.

## 2 RESULTS

### 2.1 Both ZFP541 and KCTD19 are required for male fertility

In mice, *Zfp541* and *Kctd19* are located on chromosome 7 and chromosome 8, respectively. RT‐PCR analysis showed that *Zfp541* was transcribed in both testes and ovaries, and *Kctd19* was specifically transcribed in testes but not ovaries (Figure S1). Moreover, The mRNA of *Zfp541* was detected at least from postnatal day 8 (PD8), however, two *Kctd19* mRNA variants were detected from PD14 in mouse testes (Figure S1). The single *Zfp541* transcript contained 16 exons with a 3909 bp ORF (Figure S1). The long *Kctd19* variant contained 17 exons with a full ORF of 2850 bp, the short variant lacked the 69 bp‐exon 6 and was more highly expressed than the long variant (Figure S1). ZFP541 and KCTD19 proteins were detected by Western blot in mouse testes from PD10 and PD16, respectively (Figure S1). Although there were two variants, only a single KCTD19 band was detected by Western blot^27^ (Figure S1), implying that probably only the short variant is translated given its higher abundance than the long variant. As reported, the physical interaction between ZFP541 and KCTD19 was also confirmed by testis co‐IP and yeast two‐hybrid experiments (Figure S1).

**FIGURE 1:**
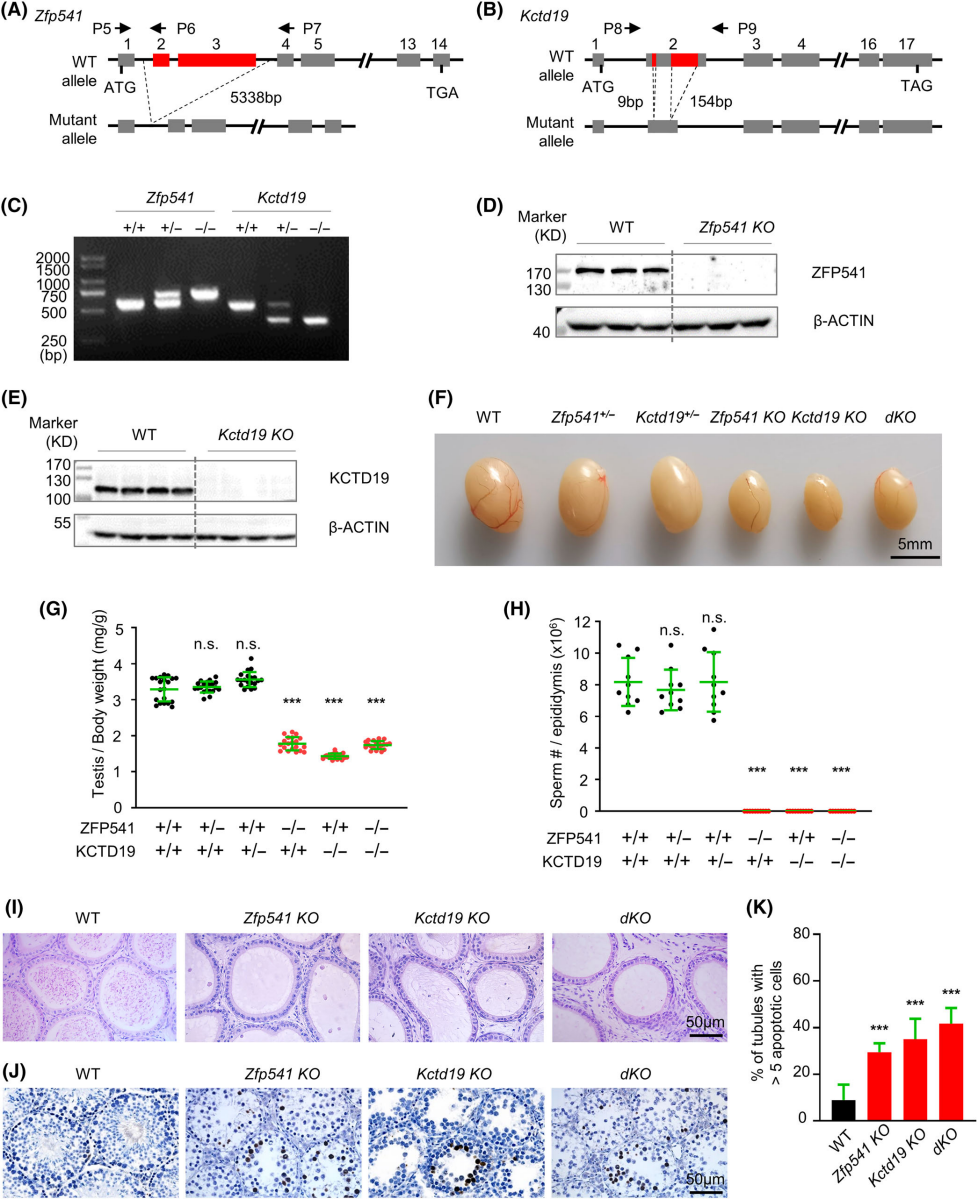
Both *Zfp541* ^*−/−*^ and *Kctd19* ^*−/−*^ male mice are sterile. (A) Schematic diagram of *Zfp541* knockout allele. Exon 2 and exon 3 are deleted in the mutant. The positions of primers P5‐P7 used for genotyping are indicated. (B) Schematic diagram of *Kctd19* knockout allele. Two DNA fragments (9 and 154 bp) within exon 2 are deleted in the mutant. The positions of primers P8 and P9 used for genotyping are indicated. (C) Genotyping to confirm *Zfp541* or *Kctd19* knockout with primers indicated in (A) and (B). (D) Western blot to confirm *Zfp541* knockout in 8‐week testis lysates using an antibody against ZFP541. *n* = 3 WT and *n* = 3 *Zfp541 KO* mice. (E) Western blot to confirm *Kctd19* knockout in 8‐week testis lysates by using an antibody against KCTD19. *n* = 4 WT and *n* = 4 *Kctd19 KO* mice. (F) Representative images to show 8‐week testes sizes of WT, *Zfp541* ^*+/−*^, *Kctd19* ^*+/−*^, *Zfp541 KO*, *Kctd19 KO*, and *dKO* mice. (G) The ratios of testis/body weight from 8‐week *Zfp541 KO*, *Kctd19 KO*, and *dKO* mice are significantly reduced compared to WT, *Zfp541* ^*+/−*^, and *Kctd19* ^*+/−*^ mice at the same age. Each dot represents the ratio of one testis to its body weight in a mouse. Error bar, mean ± SD. *n* = 10 mice for each genotype. n.s. (not significant), *p* ≥ 0.05; ***, *p* < 0.001; two‐tailed Student's t‐test. (H) ~0.8 × 10^7^ sperms are counted per epididymis from 8‐week WT, *Zfp541* ^*+/−*^ and *Kctd19* ^*+/−*^ mice, but no sperm is observed in 8‐week *Zfp541 KO*, *Kctd19 KO* and *dKO* mice. Error bar, mean ± SD. *n* = 10 mice for each genotype. n.s. (not significant), *p* ≥ 0.05; ***, *p* < 0.001. (I) No sperm is observed in 8‐week *Zfp541 KO*, *Kctd19 KO*, and *dKO* mice as revealed by HE staining in cauda epididymis. (J) A large number of apoptotic spermatocytes (dark brown) in 8‐week *Zfp541 KO*, *Kctd19 KO*, and *dKO* testes by TUNEL staining. (K) The proportion of seminiferous tubules containing more than 5 apoptotic cells. *n* = 855 (WT), 824 (*Zfp541 KO*), 855 (*Kctd19 KO*), and 796 (*dKO*) tubules; 3 mice for each genotype. Error bar, 95% confidence interval. ***, *p* < 0.001; Chi‐square test.

To explore the roles of *Zfp541* and *Kctd19* in spermatogenesis, *Kctd19* and *Zfp541* single‐knockout mice and double‐knockout mice were constructed. A 5338 bp DNA fragment containing exons 2 and 3 of *Zfp541*, a 9 bp and a 154 bp DNA fragments within exon 2 of *Kctd19* were deleted using the CRISPR/Cas9 system to generate *Zfp541* ^*−/*−^ and *Kctd19* ^*−/−*^ mice (designated as *Kctd19 KO and Zfp541 KO*), respectively (Figures 1A,B and S2). These mutant mice were confirmed by genomic PCR, RT‐PCR from testes followed by DNA sequencing, and Western blot for testis lysates (Figures 1C,E and S2). The double knockout mice (designated as *dKO*) were generated by crossing *Kctd19* ^*+/−*^ with *Zfp541* ^*+/*−^ mice. Both male and female of the two single and one double mutant mice appeared to grow and develop normally. Female mice were fertile, however, male mice were sterile for *Kctd19 KO*, *Zfp541 KO*, and *dKO* (Figure S3). Given *Kctd19* ^*+/−*^ or *Zfp541* ^*+/−*^ male mice had normal fertility, they were also used as controls as WT male mice in this study (Figure S3). The sizes of testes of 8‐week male mice for *Kctd19 KO*, *Zfp541 KO*, *and dKO* were significantly smaller than those of the control mice (Figure 1F,G). Moreover, no sperm was observed in the epididymis of 8‐week *Kctd19 KO*, *Zfp541 KO*, or *dKO* male mice, in contrast to ~0.8 × 10^7^ sperms per epididymis observed in the control mice (Figure 1H). Consistently, histological analysis with Haematoxylin and Eosin (H&E) staining identified a large number of mature spermatozoa in the cauda epididymis of WT male mice, however, no spermatozoa were observed in *Kctd19 KO*, *Zfp541 KO*, *or dKO* mice, suggesting defective spermatogenesis in these mutants (Figure 1I). Further analysis by Periodic Acid Schiff (PAS) staining of testis sections showed that, unlike WT male mice, no post‐meiotic round spermatid or elongating spermatid was found in the seminiferous tubules of testes from *Kctd19 KO*, *Zfp541 KO*, or *dKO* mice (Figure S4A). Additionally, a considerable number of tubules bearing apoptotic MI‐like spermatocytes were identified in testes from *Kctd19 KO* mice, however, apoptotic spermatocytes from pachytene to diplotene were often observed in *Zfp541 KO* and *dKO* mice, although they were rarely observed in WT mice (Figure S4). The existence of apoptotic spermatocytes was further confirmed by TUNEL staining of testis sections in these mutants (Figure 1J,K). Overall, these findings support that both KCTD19 and ZFP541 are required for normal spermatogenesis and male fertility.

### 2.2 Both ZFP541 and KCTD19 are required for the completion of male meiosis

**FIGURE 2:**
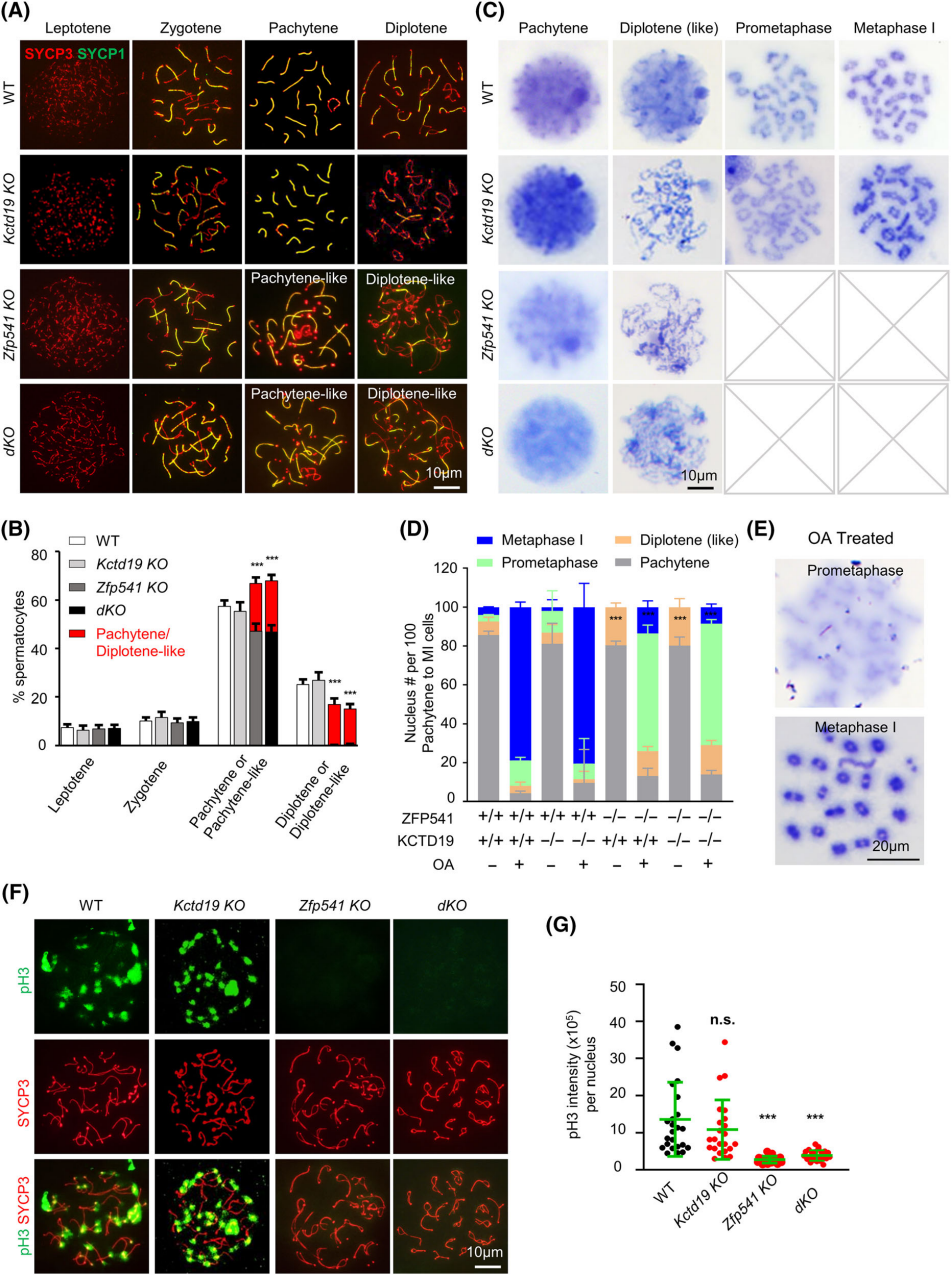
*Zfp541* ^*−/−*^ and *Kctd19* ^*−/−*^ spermatocytes show meiotic prophase I arrest. (A) Representative images to show spermatocytes in different stages of prophase I. Spread spermatocytes are immune‐stained to show SYCP3 and SYCP1. (B) Quantification of (A) to show proportions of spermatocytes in different prophase I substages. *n* = 1587, 676, 995, 1187 spermatocytes for WT, *Kctd19 KO*, *Zfp541 KO* and *dKO*, respectively; 3 mice (8‐week) for each genotype. Error bar, 95% confidence interval. ***, *p* < 0.001; Chi‐square test. (C) Chromosome spreading in combination with Giemsa staining to identify stages from pachytene to metaphase I. Pachytene, diplotene (like), pro‐metaphase, and metaphase I spermatocytes are distinguished based on chromosome condensation and morphology. Pro‐metaphase and metaphase I spermatocytes are not found in *Zfp541 KO* and *dKO* mice. (D) The proportions of spermatocytes in different stages after OA treatment. *n* = 1555, 1120, 1626, and 1365 OA‐treated spermatocytes for WT, *Kctd19 KO*, *Zfp541 KO*, and *dKO*, respectively; *n* = 1731, 1453, 2095, 1908 spermatocytes (without OA treatment) for WT, *Kctd19 KO*, *Zfp541 KO*, and *dKO*, respectively; 3 adult mice for each genotype. Error bar, 95% confidence interval; ***, *p* < 0.001 by Chi‐square test. (E) Representative Giemsa staining images to show pro‐metaphase and metaphase I nuclei after OA treatment. (F) Immunostaining of spread spermatocytes to show SYCP3 and pH 3 in late diplotene. (G) Quantification of pH 3 intensity from (F). *n* = 24, 23, 25, and 24 nuclei for WT, *Kctd19 KO*, *Zfp541 KO* and *dKO*, respectively. Three mice (8‐week) for each genotype; Error bar, mean ± SD; n.s. (not significant), *p* ≥ 0.05; ***, *p* < 0.001; two‐tailed Student's *t*‐test.

To further explore the roles of ZFP541 and KCTD19 in male meiosis, the progression of meiotic prophase I of spermatocytes from 8‐week WT, *Kctd19 KO*, *Zfp541 KO*, and *dKO* mice was examined. SYCP3 is a component of chromosome axes and SYCP1 is a central element of SC. The composition of different‐stage spermatocytes in testes was analysed by the coimmunostaining pattern of SYCP3 and SYCP1^39^ (Figure 2A). During leptotene, SYCP3 appears as dots or short lines indicating developing meiotic chromosome axes. SYCP3 assembles into continuous lines along chromosome axes and the SYCP1 signal appears between coaligned homologs to initiate synapsis during zygotene. Synapsis is completed and cells enter pachytene when SYCP1 appears along the full length of chromosome axes. When SYCP1 disassembles from the synapsed homologs, cells enter diplotene (Figure 2A). In WT and *Kctd19 KO* male mice, the proportions of spermatocytes at each stage from leptotene to diplotene were comparable and synapsis appeared to be normal (Figure 2A,B). However, in *Zfp541 K*O and *dKO* male mice, a proportion of pachytene spermatocytes with detached chromosome ends (‘pachytene‐like’; ~66.50% of late pachytene nuclei) were observed, and the sum of the pachytene and pachytene‐like spermatocytes took up a larger proportion of spermatocytes in these two mutants than that in WT or *Kctd19 KO* (Figure 2B). Consistently, fewer diplotene spermatocytes were observed, moreover, almost all these nuclei had one or more chromosomes with detached ends (‘diplotene‐like’) in these two mutants (Figure 2B).

To further examine meiosis progression from pachytene to MI, chromosome spreading in combination with Giemsa staining was performed. Spermatocytes from pachytene to MI can be distinguished according to chromosome condensation and morphology (Figure 2C). Chromatin is uncondensed at pachytene, while linear chromatin is visible when cells enter diplotene. At prometaphase I, chromosomes are condensed to be shorter and chiasmata can be clearly seen between homologous chromosomes. At metaphase I, chromosomes are further compacted to condensed bivalents, connected by more prominent chiasmata. In adult WT testes, among spermatocytes from pachytene to MI, most (~86%) were at pachytene, ~7% were at diplotene, ~3% were at pro‐MI, and ~ 4% were at MI (Figure 2D). In *Kctd19 KO* mice, the proportion of spermatocytes at different stages was comparable to WT (81%, 6%, 11%, and 2%, respectively), although there were slightly more at prometaphase I, indicating possible arrest just before MI (Figure 2C,D). However, in *Zfp541 KO* and *dKO* mice, ~80% of spermatocytes were at pachytene, the rest were at diplotene, and spermatocytes after diplotene were not observed, indicating possible arrest at diplotene (Figure 2C,D). Interestingly, a considerable number of spermatocytes in these two mutants had less compacted, ribbon‐shaped, and intermingled chromosomes, which were more likely at late diplotene (Figure 2C).

Okadaic acid (OA) is a specific protein phosphatase inhibitor, which can induce spermatocytes at or after mid‐pachytene to override cell cycle checkpoint to rapidly and prematurely enter MI.^40^ After WT and *Kctd19 KO* spermatocytes were isolated and treated by OA, ~80% of spermatocytes were at MI and ~10% were at prometaphase I (Figure 2D,E). However, ~60% of *Zfp541 KO* and *dKO* spermatocytes were at prometaphase I, and only ~10% were at MI after OA treatment (Figure 2D). The results suggest that *Kctd19 KO* spermatocytes were defective in chromosome condensation at late diplotene, arrested just before MI, and then underwent apoptosis in vivo (Figures 2D,E and S4). After in vitro OA treatment, these spermatocytes are competent to enter MI (Figure 2D,E). However, *Zfp541 KO* and *dKO* spermatocytes were defective at least at pachytene, arrested at diplotene, and then underwent apoptosis (Figures 2D,E and S4). Therefore, these spermatocytes fail to enter MI even after OA treatment (Figure 2D,E). These results further imply defective chromosome compaction/organization in these mutants and *Zfp541 KO* shows severer and earlier defects than *Kctd19 KO*. Consistently, histone H3 phosphorylation on Ser10 (pH 3), which appears from late diplotene accompanied by chromosome condensation,^41^ was clearly observed in WT and *Kctd19 KO* but not *Zfp541 KO* or *dKO* diplotene spermatocytes (Figure 2F,G).

### 2.3 Both ZFP541 and KCTD19 are required for normal meiotic recombination

**FIGURE 3:**
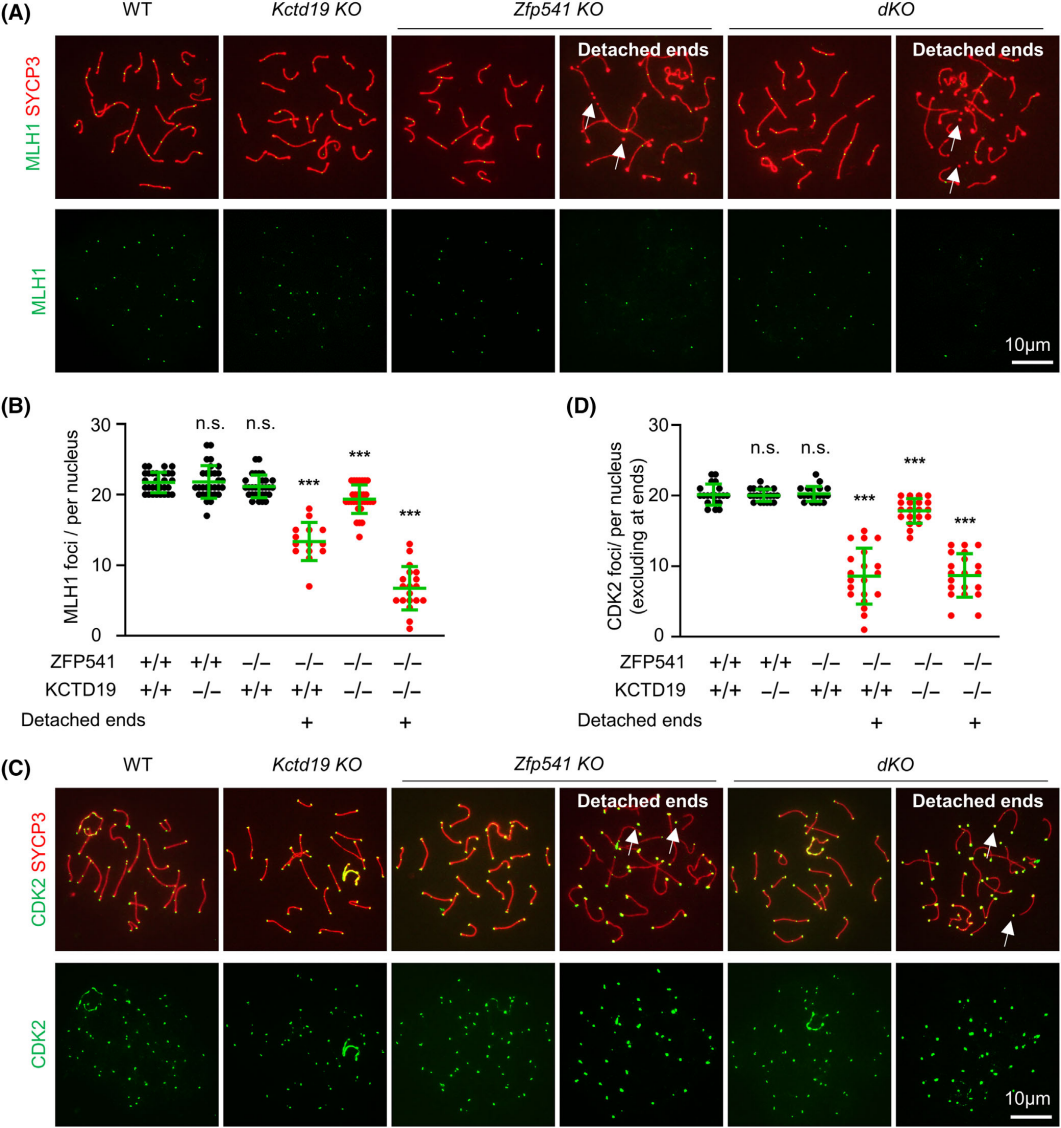
*Kctd19* ^*−/−*^ *Zfp541* ^*−/−*^ mice show defective crossover recombination. (A) Representative images to show MLH1 foci in pachytene spermatocytes. White arrows, detached chromosome ends. (B) Quantification of MLH1 foci per nucleus from (A). *n* = 30 morphologically normal pachytene nuclei (three 8‐week mice) for each genotype. *n* = 14 and 19 pachytene nuclei with detached ends for *Zfp541 KO* and *dKO*, respectively. Error bar, mean ± SD. n.s. (not significant), *p* ≥ 0.05; ***, *p* < 0.001; two‐tailed Student's t‐test. (C) Representative images to show CDK2 foci in pachytene spermatocytes. (D) Quantification of CDK2 foci per nucleus from (C). *n* = 20 morphologically normal pachytene nuclei (three 8‐week mice) for each genotype. *n* = 20 pachytene nuclei with detached ends for *Zfp541 KO* and *dKO*, respectively. Error bar, mean ± SD. n.s. (not significant), *p* ≥ 0.05; ***, *p* < 0.001; two‐tailed Student's *t*‐test.

Meiotic recombination is a central event of meiosis. MLH1 mediates the resolution of recombination intermediate dHJs to produce mature COs, which is required for faithful homologs segregation. Thus, the MLH1 focus is commonly used as a reliable CO marker.^18^, ^42^, ^43^ Immunostaining of MLH1 showed that there were an average of ~22 MLH1 foci per pachytene nucleus in WT and *Kctd19 KO* (Figure 3A,B). Consistently, similar numbers of chiasmata were observed in OA‐induced MI nuclei in WT and *Kctd19 KO* (21.86 ± 0.73 vs. 21.74 ± 0.75, mean ± SD). These results confirm that *Kctd19 KO* spermatocytes have normal meiotic recombination. In *Zfp541 KO*, morphologically normal pachytene nuclei showed the WT‐level MLH1 foci, however, those nuclei with detached chromosome ends showed fewer MLH1 foci (Figure 3A,B). In *dKO*, all pachytene spermatocytes showed fewer MLH1 foci, although those morphologically abnormal nuclei showed much fewer MLH1 foci (Figure 3A,B). At pachytene, CDK2 localizes on CO sites and also at chromosome ends. Therefore, the number of interstitial CDK2 foci reflects the CO level^44^ (Figure 3C). Immunostaining of CDK2 confirmed the results from MLH1 foci (Figure 3C,D). These results suggest that ZFP541 is required for normal CO formation and KCTD19 probably also plays a minor and redundant role in this process. Therefore, *Kctd19 KO* spermatocytes show no obvious defect in CO formation, however, *Zfp541 KO* and *dKO* spermatocytes show decreased CO levels.

COs derived from programmed DSBs. γH2AX and RPA are two commonly used markers for DSB generation and repair, and thus indicators of meiotic recombination progression.^45^, ^46^, ^47^ The γH2AX and RPA signal appeared during leptotene and gradually increased and reached a maximum at zygotene. After that, accompanied by DSB repair, the number of RPA foci gradually decreased to a rarely observable level at late pachytene and completely disappeared at diplotene (Figure S5). The γH2AX signal on autosomes dramatically decreased, however, the bright signal appeared on the XY body at pachytene. At diplotene, the γH2AX signal was rarely detected on autosomes but the strong signal was maintained on the XY body (Figure S5). When examined, the pattern and dynamics of γH2AX and RPA were comparable in WT and *Kctd19 KO* spermatocytes (Figure S5). These results in combination with the normal CO number suggest that KCTD19 is not required for DSB formation and repair, CO formation, or meiosis progression to diplotene in spermatogenesis. Thus, *Kctd19 KO* is not further examined in meiotic recombination. *Zfp541 KO* and *dKO* also showed normal patterns and dynamics of γH2AX from leptotene to mid‐pachytene and the recurrence of γH2AX was observed from late pachytene as previously reported (Figure S6A,B). Given the highly similar phenotypes between *Zfp541 KO* and *dKO*, the recombination process was only examined in detail in *dKO* in the following experiments.

After DSB end resection, RPAs bind to ssDNA ends. DMC1/RAD51 then replaces RPAs to promote DSB repair using homologs as templates and the formation of D‐loops. It has been proposed that the MZIP2‐TEX11‐SPO16 complex in mice (the Zip2‐Zip4‐Spo16 complex in budding yeast) binds and stabilizes probably D‐loops.^30^, ^48^, ^49^, ^50^, ^51^, ^52^, ^53^, ^54^ The MSH4‐MSH5 complex then promotes and stabilizes double Holliday junctions.^55^, ^56^, ^57^ Immunostaining of RPA, RAD51, DMC1, TEX11, and MSH4, revealed that each of them showed similar dynamics between WT and *dKO* from leptotene to mid‐pachytene (Figures S6C–H and S7). However, late pachytene spermatocytes in *dKO* showed significantly higher numbers of foci than WT (Figures S6C–H and S7). This is probably due to the recurrence of DSBs at late pachytene although it is also possible that there are minor DSB repair defects in *dKO* mice.

**FIGURE 4:**
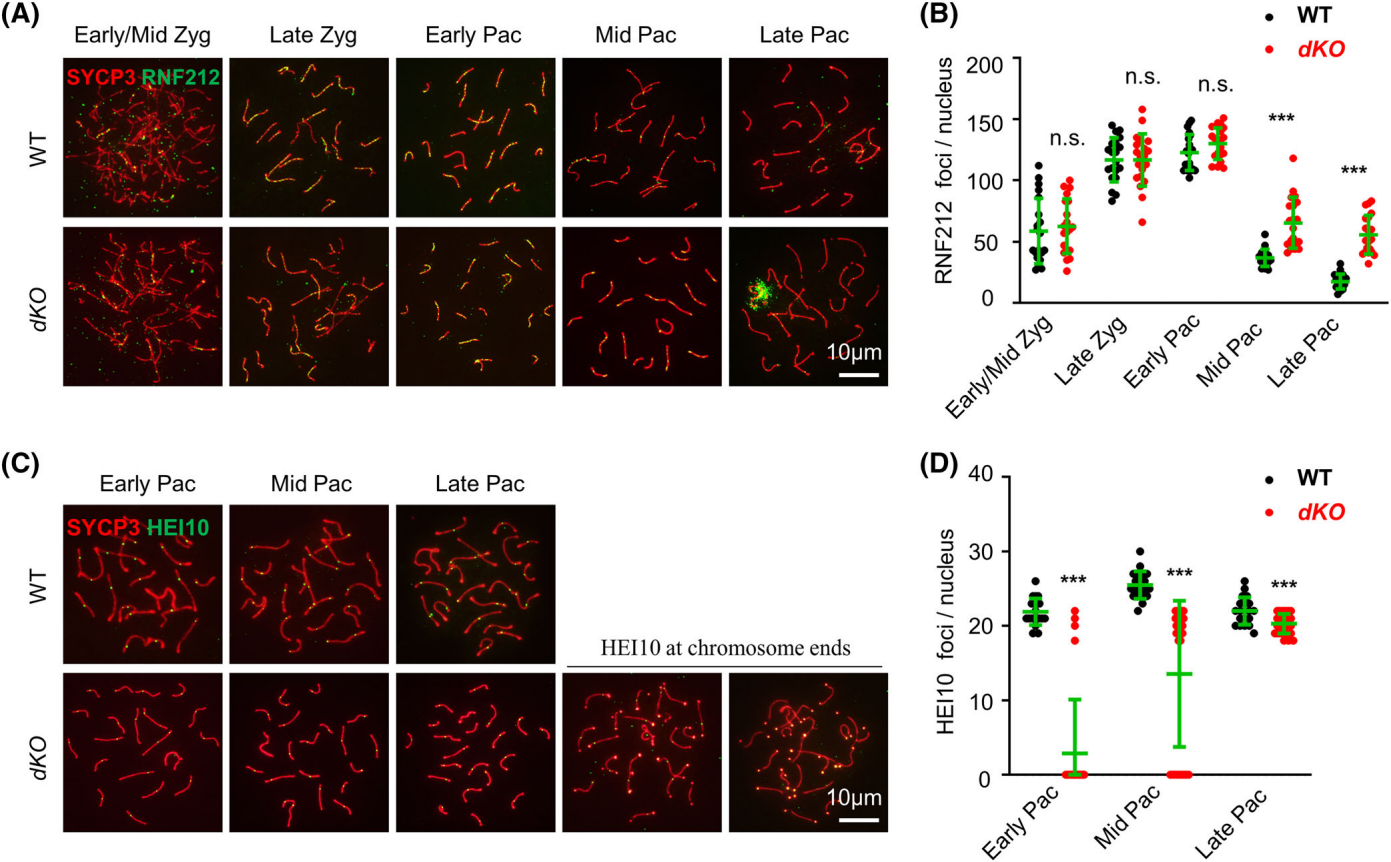
*Kctd19* ^*−/−*^ *Zfp541* ^*−/−*^ mice show defective chromosome distribution of RNF212 and HEI10 foci. (A) Representative images to show RNF212 in spermatocytes. (B) Quantification of RNF212 foci per nucleus from (A). Several late pachytene nuclei have a big patch of RNF212 around XY chromosomes. *n* = 20 for each genotype at each stage. Error bar, mean ± SD. n.s. (not significant), *p* ≥ 0.05; ***, *p* < 0.001; two‐tailed Student's *t*‐test. (C) Representative images to show HEI10 in spermatocytes. Some (32%) late pachytene nuclei show chromosome end located HEI10 foci in *Kctd19* ^*−/−*^ *Zfp541* ^*−/−*^. (D) Quantification of HEI10 foci per nucleus from (C). *n* = 20 for WT and *n* = 30 for the mutant at each stage. Error bar, mean ± SD. ***, *p* < 0.001; two‐tailed Student's *t*‐test.

The SUMO ligase RNF212 is required to stabilize the MSH4‐MSH5 and MZIP2‐TEX11‐SPO16 complex and collaborates with the ubiquitin ligase HEI10 to promote CO formation.^58^, ^59^, ^60^ Further examination showed that *dKO* had more RNF212 foci at mid and late pachytene (Figure 4A,B). The striking recombination defect in *dKO* was observed by examination of HEI10: (1) most (85.71%, *n* = 28) early pachytene nuclei and a significant proportion (32.5%, *n* = 40) of mid pachytene nuclei did not have any HEI10 foci, (2) late pachytene nuclei showed fewer HEI10 foci, and (3) more interestingly, in several late pachytene nuclei, most HEI10 foci located at/near chromosome ends (Figure 4C,D). These results suggest that defective recruitment of HEI10 to chromosomes and recombination defects can be observed at least from early pachytene in *dKO*. Since chromosome ends located MLH1 foci were rarely observed and fewer MLH1 foci were observed than HEI10 foci, it is likely that some HEI10 foci are mislocated on chromosomes and/or some HEI10 foci fail to recruit foci for the formation of crossovers. This idea seems to be consistent with the previous proposal that HEI10 works as a structure‐based signal transduction protein to temporally and spatially integrate information from the structural SC and the associated recombination complexes.^61^

### 2.4 ZFP541 controls a few transcription factors to regulate the transcription program

**FIGURE 5:**
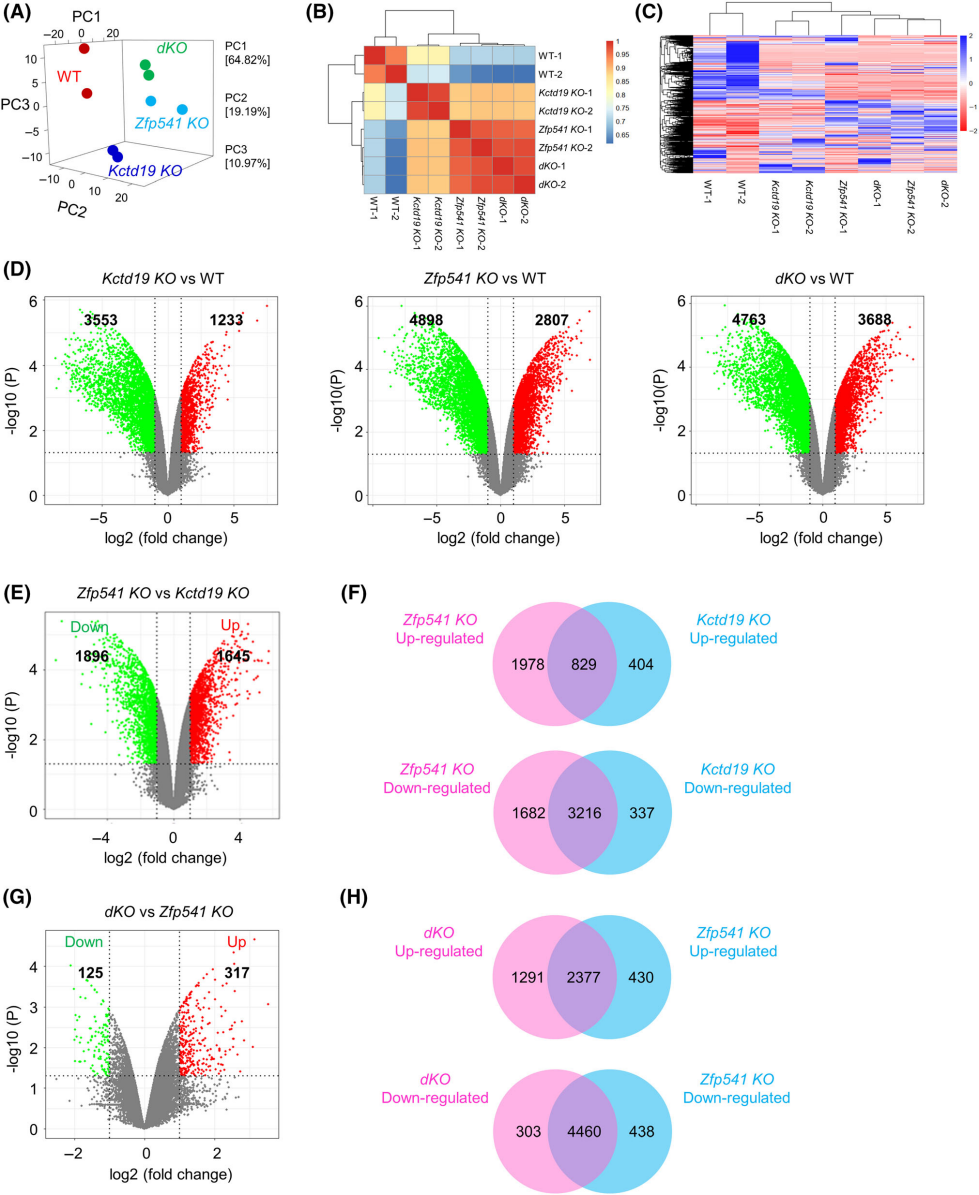
RNA‐seq analyses indicate ZFP541 and KCTD19 regulate transcription in the same pathway. (A) Principal component analyses (PCA) of RNA‐seq data to show *Zfp541 KO* is close to *dKO*. (B) Heatmap to show the Pearson correlation among RNA‐seq data. (C) The heatmap of DEGs among WT, *Kctd19 KO*, *Zfp541 KO*, and *dKO*. (D) Volcano plots to show DEGs in each mutant compared with WT. The difference with *p* < 0.05 and |log2(fold change)| ≥ 1 is considered to be significant. The red and green spots show up‐regulated and down‐regulated DEGs, respectively. The grey spots indicate differences are not significant. (E) Volcano plot to show DEGs in *Zfp541 KO* compared with *Kctd19 KO*. (F) Venn plot to show the shared and distinct DEGs between *Zfp541 KO* and *Kctd19 KO*. (G) Volcano plot to show DEGs in *dKO* compared with *Zfp541 KO*. (H) Venn plot to show the shared and distinct DEGs between *dKO* and *Zfp541 KO*.

Given previously reported roles for ZFP541 and KCTD19 in regulating transcription, RNA‐seq (RNA‐sequencing) with pachytene spermatocytes was done in parallel for WT, *Kctd19 KO*, *Zfp541 KO*, and *dKO*. The results of two experiments for each genotype showed good repeatability (Figure 5A–C). As reported and consistent with the phenotypes of the two single and one double mutants, the results were obtained (Figure 5D–H and S8). (1) Compared with WT, more DEGs (differentially expressed genes) were found in *Zfp541 KO* than in *Kctd19 KO* (Figure 5D). (2) The numbers of DEGs in *dKO* were similar to that in *Zfp541 KO* (Figure 5D). (3) For each mutant, more down‐regulated DEGs were observed than up‐regulated DEGs (Figure 5D). (4) A majority of DEGs in *Kctd19 KO* were also observed in *Zfp541 KO* (Figures 5E,F and S8). (5) Almost all DEGs in *Zfp541 KO* were also observed in *dKO* (Figure 5G,H and S8). These results suggest that ZFP541 works upstream of KCTD19 and plays a major role, however, KCTD19 only plays a minor and probably redundant role in regulating gene expression. Interestingly, GO‐BP (GO terms analysis of biological process) analysis showed that up‐regulated DEGs were enriched in chromatin organization/histone modification, recombination, and cell cycle, however, down‐regulated DEGs were enriched in gamete generation and development in all three mutants (Figure S8).

**FIGURE 6:**
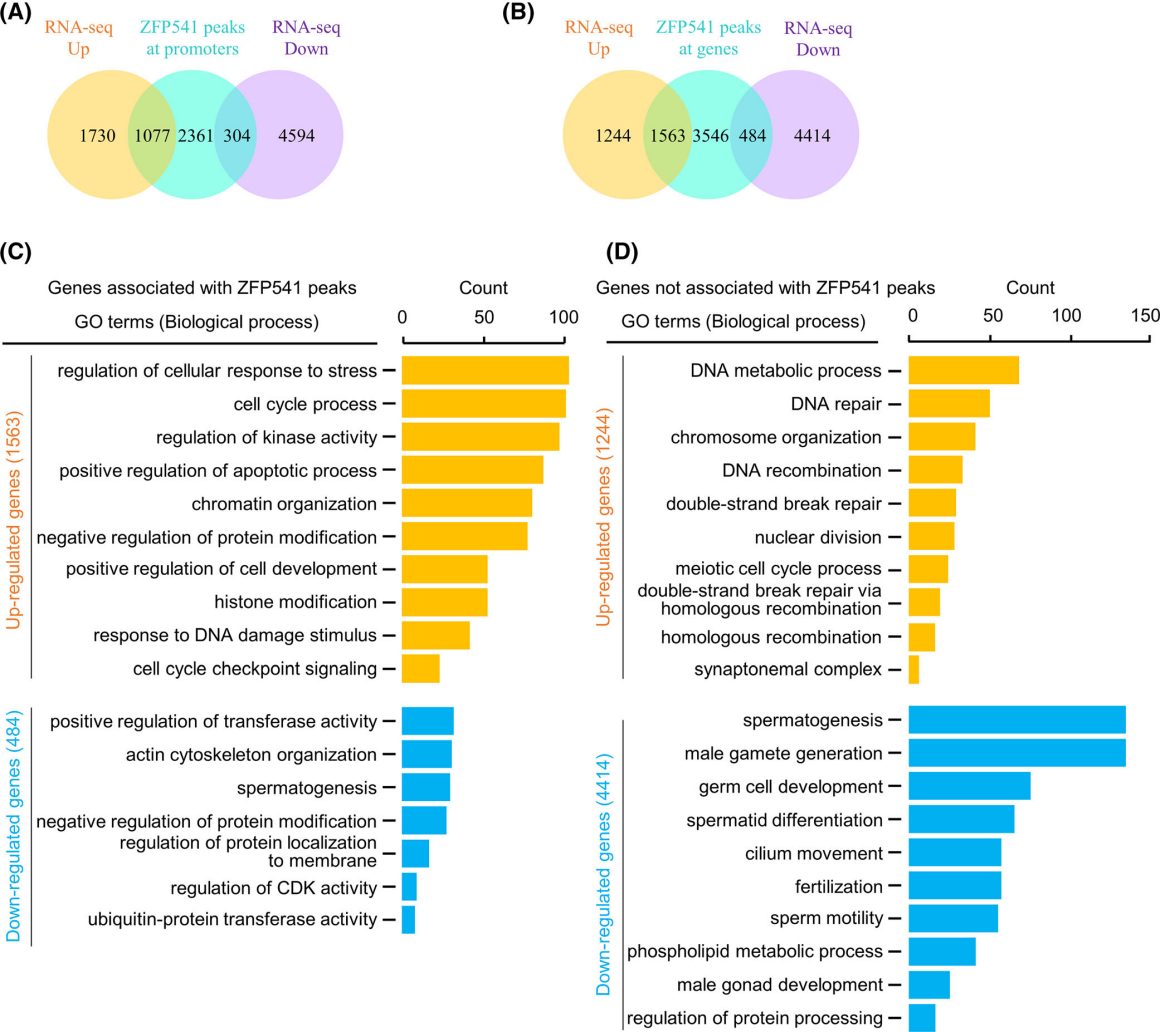
RNA‐seq in combination with ChIP‐seq analyses to identify genes directly regulated by ZFP541. (A) Venn plot to show promoters of DEGs associated with ZFP541. (B) Venn plot to show DEGs (either at promoters or gene bodies) associated with ZFP541. (C) GO enrichment analysis of DEGs associated with ZFP541. Genes involved in the processes of chromatin organization/histone modification and cell cycle are enriched. (D) GO enrichment analysis of DEGs not associated with ZFP541. Genes involved in the processes of meiotic recombination and late meiosis are enriched.

Since *Zfp541 KO* and *dKO* show very similar phenotypes and transcription profiles, combined analysis of ZFP541 RNA‐seq and ChIP‐seq data was performed to further understand its roles in transcription. As previously reported, 90.46% (7450 among 8236) of ZFP541 binding peaks on genomic DNA were tightly associated with 5593 genes (4956 peaks located on promoters of 3742 genes and 2494 peaks located on bodies of 1851 genes), and the rest, a minor proportion (786 peaks, 9.54%), located on regions far away from any known or predicted genes. Further analysis showed that only 1077 of 2807 up‐regulated DEGs and 304 of 4898 down‐regulated DEGs were associated with ZFP541 on promoters (Figure 6A). This means that only 38.37% (1077 among 2807) of up‐regulated and 6.21% (304 among 4898) of down‐regulated DEGs, that is, a total of 17.92% (1381 among 7705) of DEGs, may be considered and interpreted as directly regulated by ZFP541. It is also possible that ZFP541 binding on gene bodies to regulate transcription. If so, 55.68% (1563 among 2807) of up‐regulated and 9.88% (484 among 4898) of down‐regulated DEGs, that is, total 26.57% (2047 among 7705) of DEGs, can be directly regulated by ZFP541 (Figure 6B). However, the regulation mechanism of the majority (73.43%) of DEGs, including 44.32% of up‐regulated DEGs and 90.12% of down‐regulated DEGs, is still unknown. GO‐BP analysis showed that (1) DEGs associated with and probably directly regulated by ZFP541 were enriched in the processes of cell cycle and chromatin organization/histone modification (Figure 6C), however, (2) DEGs not associated with and thus indirectly regulated by ZFP541 were involved in meiosis, more specifically, up‐regulated DEGs were enriched in meiotic recombination and down‐regulated DEGs were enriched in late meiosis and sperm generation/function (Figure 6D). This result raises the interesting possibility that the direct and primary role of ZFP541 is to regulate the expression of chromatin organization and cell cycle‐related genes that then further regulate the expression of genes required for meiosis (e.g., meiotic recombination).

**FIGURE 7:**
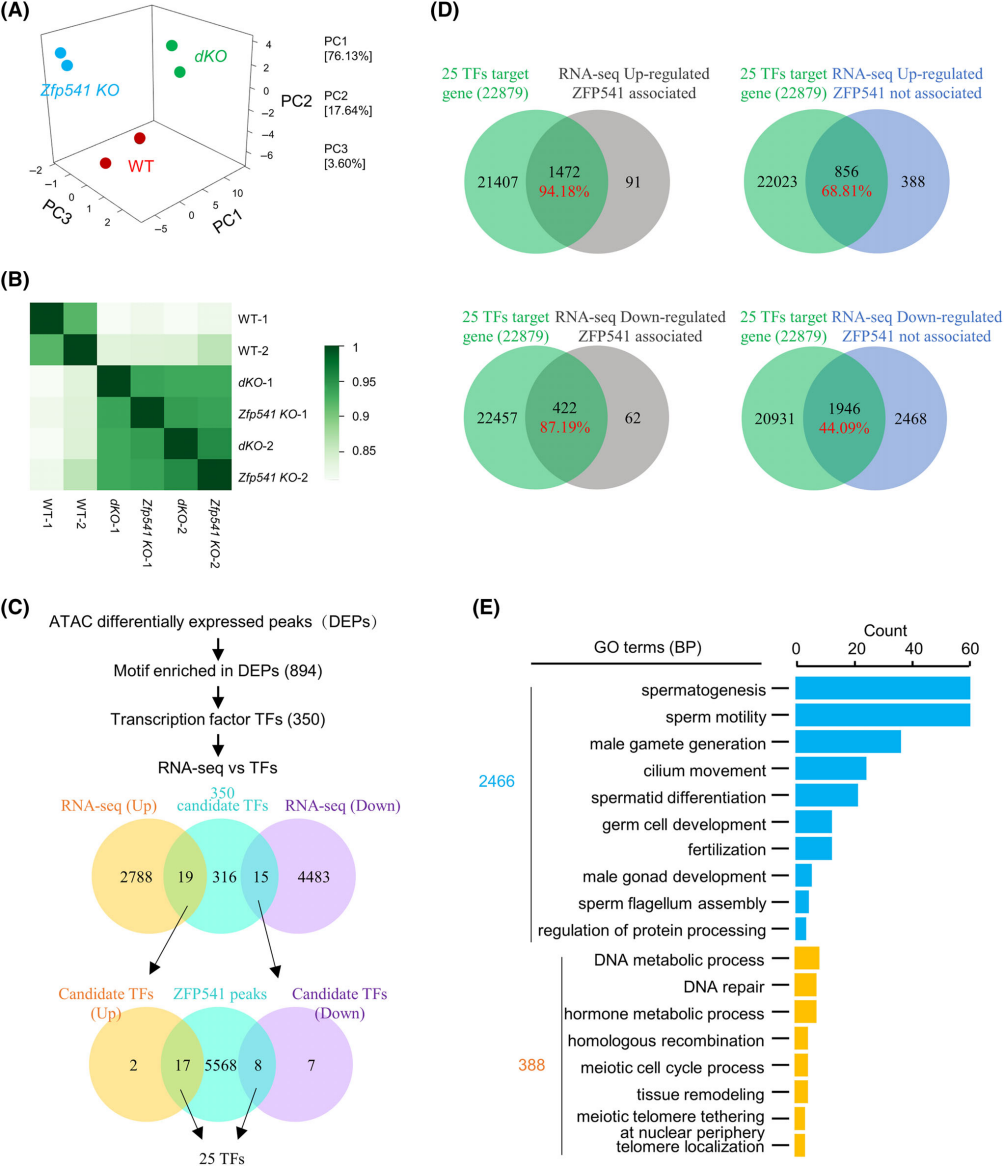
ATAC‐seq in combination with RNA‐seq analyses to identify potential TFs regulated by ZFP541. (A) Principal component analyses of ATAC‐seq data to show the repeatability between two experiments. (B) Heatmap to show the Pearson correlation among ATAC‐seq data. (C) Flow chart to show the strategy to identify candidate TFs. (D) Venn plot to show the overlap of DEGs from RNA‐seq and predicted targets of the identified 25 TFs. (E) GO enrichment analysis of DEGs that are down‐regulated but not directly associated with ZFP541.

To further investigate how the majority of DEGs are regulated and whether ZFP541 modulates chromatin organization, ATAC‐seq (assay for transposase accessible chromatin using sequencing) experiments with pachytene spermatocytes were performed. The results of two experiments for each genotype showed good repeatability (Figure 7A,B). Compared with WT, 15275 and 2851 regions were identified with increased and decreased chromatin accessibility in *Zfp541 KO*, respectively; 14367 and 1254 (total 15621) regions were identified with increased and decreased chromatin accessibility in *dKO*, respectively. Moreover, regions with altered chromatin accessibility largely overlapped between *Zfp541 KO* and *dKO* (Figure S9A). These results further indicate the similarity between *Zfp541 KO* and *dKO*. To identify transcription factors (TFs) that couple differential chromatin accessibility with gene expression, 894 motifs, corresponding to 350 TFs, were identified by Homer analysis^62^ (Figure 7C). Among these 350 possible TFs, 19 and 15 were identified with increased and decreased expression from the RNA‐seq data, respectively. Further analysis showed that a total of 25 (17 up‐regulated and 8 down‐regulated) TFs were associated with and probably directly regulated by ZFP541 (Figure 7C). Analysis with GTRD (Gene Transcription Regulation Database) predicted that the 25 TFs contributed to at least 60.95% of total DEGs, including 94.18% of ZFP541 associated and up‐regulated DEGs, 87.19% of ZFP541 associated and down‐regulated DEGs, 68.81% of ZFP541 un‐associated and up‐regulated DEGs, and 44.09% of ZFP541 un‐associated and down‐regulated DEGs (Figures 7D and S10). This analysis also showed that each of the 17 among the 25 TFs had a significant contribution although the other 8 TFs actually contributed little (Figure S10). For DEGs not regulated by these TFs, almost all (94.91%) were also not associated with ZFP541. These DEGs probably result from arrested cell cycle progression, and as expected, GO‐BP analysis showed they were enriched in the processes of sperm formation/function (Figure 7E). These results support the idea that ZFP541 directly regulates the expression of (1) a few TFs, which further regulate the expression of a large number of genes required for meiosis, and (2) chromatin organization and cell cycle‐related genes, which regulate meiotic progression and the expression of late genes. Alterations in chromatin organization and the expression of TFs could also affect each other to further regulate the progression of meiosis.

### 2.5 ZFP541 modulates chromatin organization in male meiosis

**FIGURE 8:**
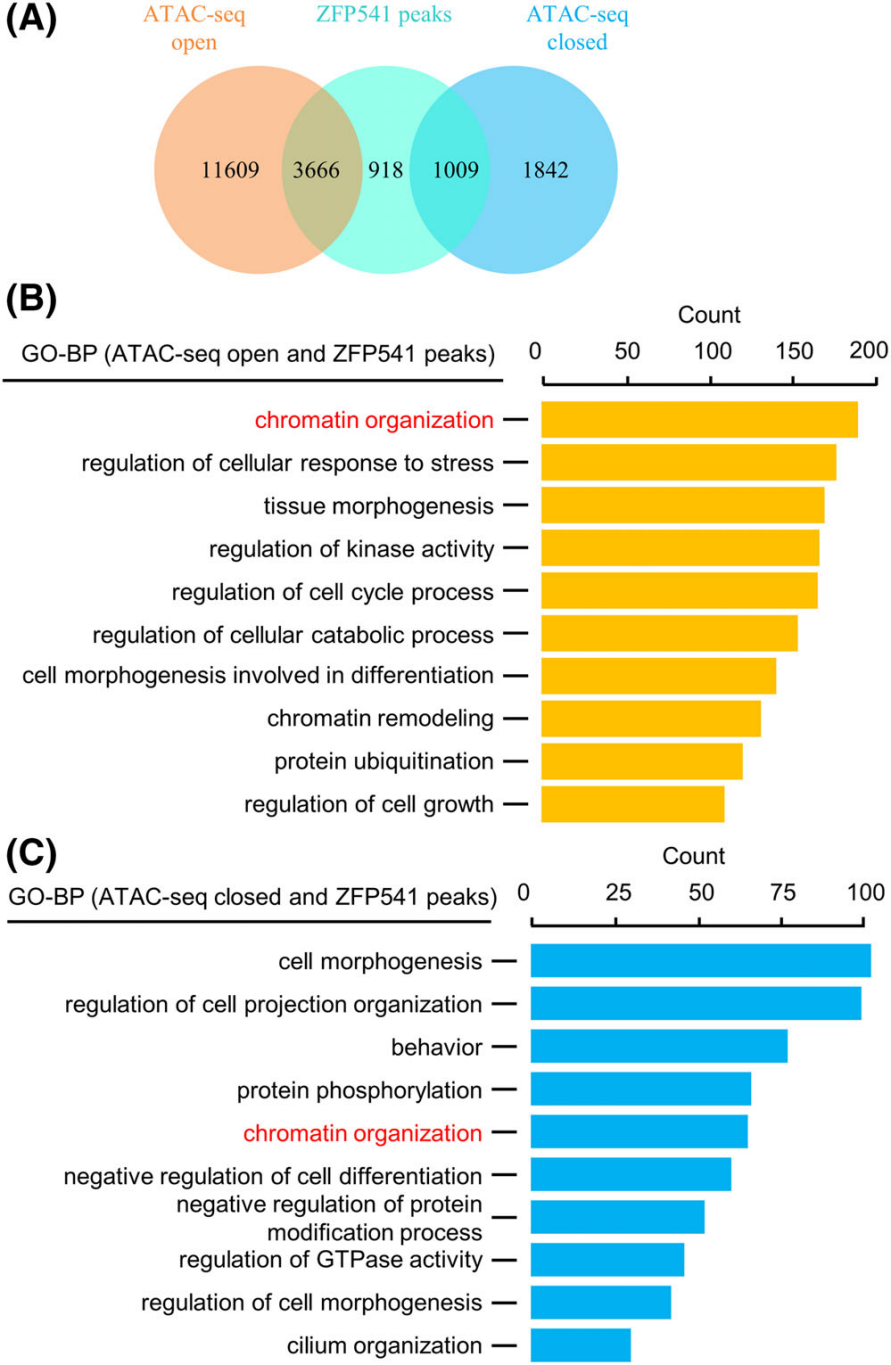
ZFP541 binding regions show altered chromatin accessibility. (A) Venn plot to show most ZFP541 binding regions overlapped with altered chromatin accessible regions. (B, C) GO enrichment analysis of overlapped regions in (A) to show chromatin organization/remodelling‐related genes are enriched in these regions.

As analysed above, most ZFP541 directly regulated DEGs were involved in the regulation of cell cycle and chromatin organization, suggesting its possible function in modulating chromatin organization (Figure 6C). Consistently, among 5593 ZFP541 binding genes, only 1563 and 484 genes (27.95% and 8.65%) showed up‐regulated and down‐regulated expression, respectively. However, most ZFP541 binding genes did not show altered expression (Figure 6B), further indicating regulating transcription may not be the major role of ZFP541. Moreover, most (76.12%) regions with altered chromatin accessibility did not affect gene expression or were not associated with any genes (Figure S9B). Furthermore, combined analysis of ATAC‐seq and ChIP‐seq data showed that most (83.59%) ZFP541 binding regions showed altered chromatin accessibility, more specifically, 65.55% (3666 among 5593) with increased chromatin accessibility and 18.04% (1009 among 5593) with decreased chromatin accessibility (Figure 8A). It is interesting to note that chromatin organization‐related genes were enriched in ZFP541 binding and chromatin accessibility altered regions (ATAC‐seq and ChIP‐seq overlapped regions) (Figure 8B,C).

**FIGURE 9:**
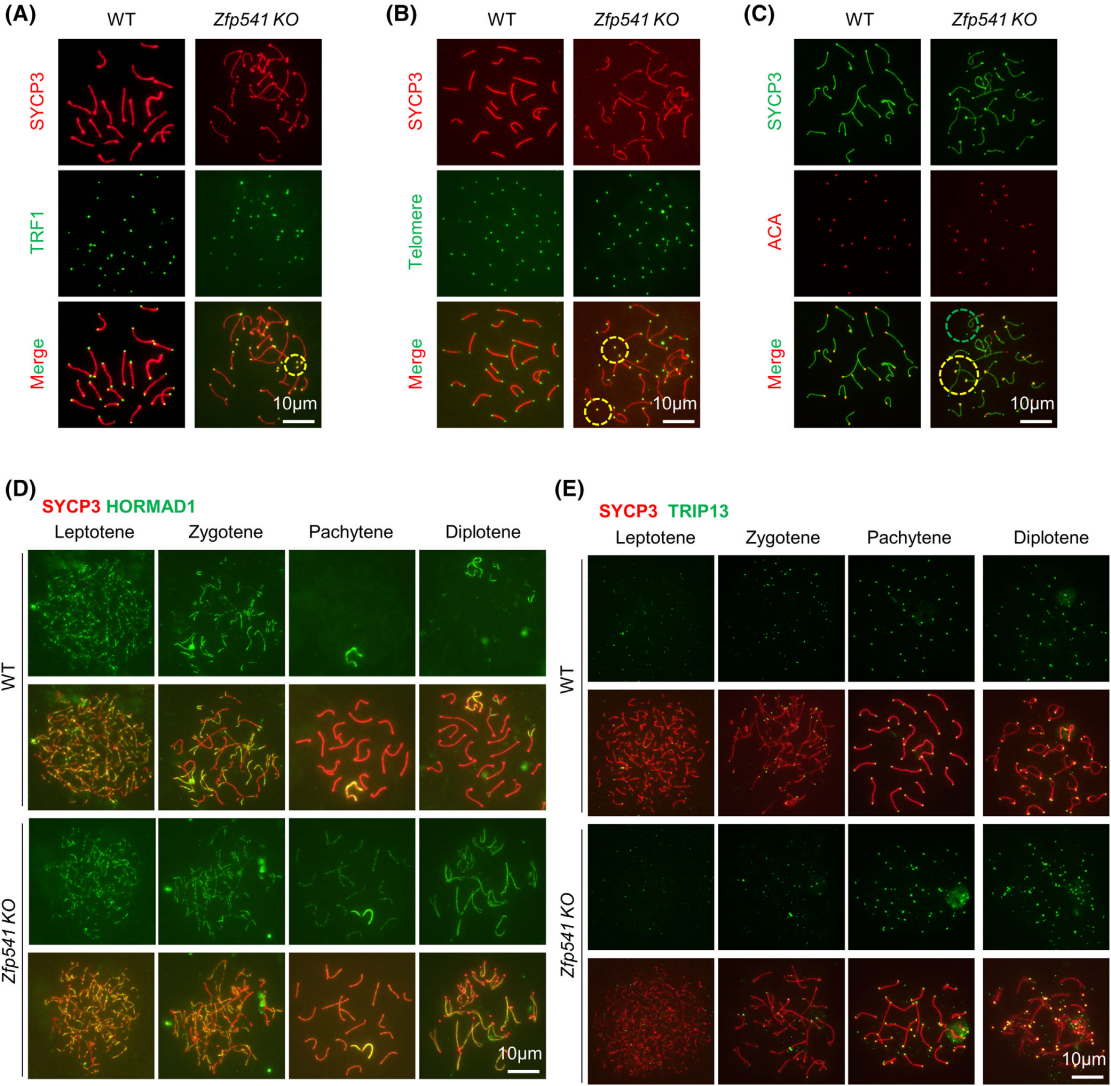
*Zfp541* ^*−/−*^ spermatocytes show aberrant chromatin organization. (A–C) The detached chromosome ends in *Zfp541 KO* pachytene spermatocytes. (A) Immunostaining to visualize telomere binding TRF1. (B) FISH with telomeric repeat DNA probe to visualize telomeres. (C) Immunostaining with anti‐centromere antibody ACA to show centromeres. (D, E) Representative images to show HORMAD1 (D) and TRIP13 (E) in spread spermatocytes.

The idea that ZFP541 modulates chromatin organization is also consistent with the existence of detached chromosome ends in a large proportion (66.50%) of late pachytene and nearly all diplotene spermatocytes in *Zfp541 KO* and *dKO*. Detached chromosome ends were further confirmed by the immunostaining of telomeric binding protein TRF1 and FISH with the telomeric repeat DNA sequence as the probe (Figure 9A,B). Given centromeres are located close to one end of chromosomes (acrocentric chromosomes) in mice, immunostaining with centromeric‐specific antibody ACA showed that detached ends could be the centromeric or the other end (Figure 9C). HORMAD1 is an important axis component and regulates DSB formation and the subsequent recombination process. HORMAD1 appears on the axis as foci at leptotene. During zygotene, bright HORMAD1 signal is on asynapsed axes and synapsed regions have only very weak signal. During pachytene, the HORMAD1 signal is barely detectable except on asynapsed regions of XY chromosomes. During diplotene, the HORMAD1 signal re‐appears on desynapsed axes^8^, ^63^, ^64^ (Figure 9D). In *Zfp541 KO*, a similar HORMAD1 staining pattern was observed as WT before pachytene, however, HORMAD1 staining was still obviously observed along fully synapsed chromosome axes at pachytene and desynapsed regions at diplotene although its intensity seems to be slightly a little bit weaker on synapsed regions (Figure 9D). TRIP13, an AAA‐ATPase, removes HORMAD1 from synapsed chromosome regions.^8^, ^63^, ^64^ However, paradoxically, increased TRIP13 was observed on chromosomes from pachytene to diplotene (Figure 9E). These results further confirm that ZFP541 is required for proper chromatin organization and distribution of chromosome axis components.

**FIGURE 10:**
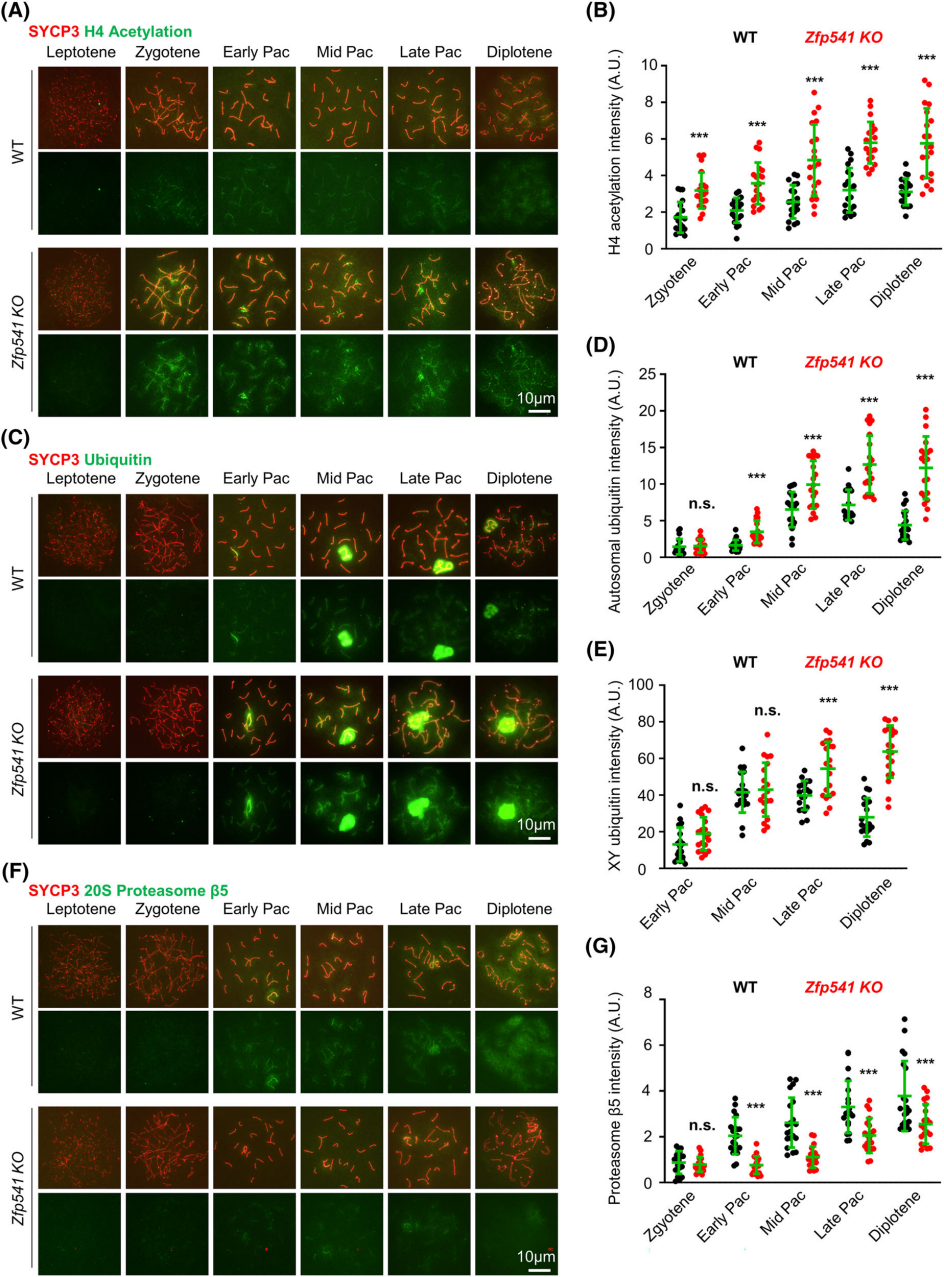
ZFP541 regulates chromatin modification. (A, B) Representative images (A) and quantification (B) to show histone H4 acetylation in spread spermatocytes. *n* = 20 nuclei for each genotype at each stage; Error bar, SD; ***, *p* < 0.001 by two‐tailed student's *t*‐test. (C‐E) Representative images (C) and quantification to show ubiquitination on autosomal (D) and XY (E) chromosomes in spread spermatocytes. *n* = 20 nuclei for each genotype at each stage; Error bar, SD; n.s. (not significant), *p* ≥ 0.05; ***, *p* < 0.001 by two‐tailed student's *t*‐test. (F, G) Representative images (F) and quantification (G) to show 20S proteasome β5 in spread spermatocytes. *n* = 20 nuclei for each genotype at each stage; Error bar, SD; n.s. (not significant), *p* ≥ 0.05; ***, *p* < 0.001 by two‐tailed student's *t*‐test.

The ZFP541‐KCTD19 complex can interact with histone deacetylase HDAC1 and HDAC2.^27^, ^28^, ^29^, ^30^ Consistent with their possible role in regulating histone deacetylation, ZFP541 began to be observed in leptotene/zygotene spermatocytes by immunostaining and *Zfp541 KO* spermatocytes showed increased histone H4 acetylation from zygotene^30^ (Figures 10A,B and S11). Further investigation showed that the ubiquitination level was increased and the localization of proteasomes on chromosomes was decreased from early pachytene (Figure 10C–G). Histone acetylation and ubiquitination/SUMOylation play important roles in multiple processes in meiosis, especially in chromatin organization and recombination.^58^, ^59^, ^60^, ^65^, ^66^ Our investigation also showed increased SUMOylation in *Zfp541 KO* spermatocytes from mid‐pachytene (Figure S12). Thus, ZFP541 probably collaborates with HDAC1/2 to modulate histone acetylation, which further affects other histone modifications and chromatin organization.

Chromatin is organized as an array of loops that are attached to their base by a loop extrusion process. The size of loops is largely determined by anchors that block the extrusion process and frequently locate at the loop base.^34^, ^35^ A large fraction of ZFP541 was found to be colocalized with the putative chromatin anchor CTCF and the cohesin subunit Rad21^8^, ^67^ (Figure S13). This raises the possibility that a number of ZFP541 localizes at anchor sites and modulates chromatin organization either directly or indirectly by regulating localizations of anchor proteins. Collectively, our above analyses suggest that ZFP541 has an important role in organizing chromatin during male meiosis.

## 3 DISCUSSION

Parallel analysis of *Kctd19 KO*, *Zfp541 KO*, and *dKO*, reveal that ZFP541 and KCTD19 work in the same genetic pathway to regulate transcription program and chromatin organization, which further affect meiotic recombination and cell cycle. We propose that ZFP541/ KCTD19 probably couple the transcription program with chromatin organization in regulating multiple events for their coordinated progression in male meiosis.

Previous studies have the argument that ZFP541/KCTD19 works primarily as an activator or a repressor to modulate transcription based on the altered expression profile and its binding at promoters.^28^, ^30^, ^31^ Our current study suggests that ZFP541/KCTD19 regulates the expression of several important transcription factors, that further regulate the expression of many downstream target genes. As discussed below, the role of ZFP541/KCTD19 in modulating chromatin organization should also contribute to the transcription program.

Transcription factors can directly modulate chromatin organization via the transcription process.^36^, ^37^, ^38^ Our study in combination with previous studies provides evidence from both cytological and molecular levels for the proposal that ZFP541/KCTD19 can also directly regulate chromatin organization during male meiosis. (1) These mutants show aberrant chromosome structure/morphology from pachytene, such as detached chromosome ends, SYCP3 polycomplex, and SYCP1 lost at chromosome ends^28^, ^29^, ^30^, ^31^ (Figures 2 and 9). These phenotypes also suggest possible early defects and are consistent with the result that pachytene nuclei cannot transit to metaphase I with OA treatment (Figure 2C–E). (2) Recurrent of DSBs and re‐appearance of recombination‐related proteins on chromosomes after mid‐pachytene,^29^, ^31^ the aberrant recruitment/localization of recombination‐associated RNF212 and HEI10 (Figure 4), the existence of two groups of pachytene nuclei with different levels of CO‐associated MLH1 and CDK2 foci^28^ (Figure 3), and more importantly, the aberrant localization of axis related proteins, such as IHO1, HORMAD1, and TRIP13 (Figure 9). (3) ZFP541/KCTD19 interacts with histone deacetylase HDAC1/2 to reduce histone acetylation level (Figure 10A,B), which could contribute to more compacted chromatin.^68^, ^69^ The globally altered ubiquitination and sumoylation might be the consequence of changed histone acetylation and chromatin organization. (4) Chromatin organization‐related genes are enriched in ZFP541 directly regulated DEGs (Figure 6C), and most ZFP541 binding regions show altered chromatin accessibility although only a small fraction of them is associated with altered gene expression (Figure 8A). (5) Most ZFP541 binding regions are also the binding regions of putative chromatin anchor CTCF and cohesin subunit RAD21 (Figure S13).

Based on the role in modulating chromatin organization, the roles of ZFP541/KCTD19 in multiple processes (the multiple phenotypes) can be easily integrated into a coherent model. ZFP541/KCTD19 binds on chromatin and plays two roles: (1) directly regulates the expression of a small number of genes, including several major TFs, which further regulate the expression of many target genes, and (2) modulates chromatin organization at many binding sites. Both roles probably are executed by the interaction with HDAC1/2. These two roles can also affect each other and work coordinately to regulate transcription programs, chromatin organization, meiotic recombination, and cell cycle. For example, genes involved in the processes of chromatin organization, recombination, and cell cycle are enriched among those regulated by ZFP541/KCTD19, and altered chromatin organization can affect the transcription, meiotic recombination, and cell cycle. Therefore, ZFP541/KCTD19 probably links chromatin organization and transcription together to tightly coordinate the progression of multiple events in meiosis: transcription, chromatin organization, meiosis‐specific events (recombination, synapsis, and desynapsis), and cell cycle.

Given ZFP541 is also expressed in the female fetal ovary, if ZFP541/KCTD19 is so important as above discussed, why their deficiency does not impair female fertility? This could be related to the differential meiosis processes between males and females. Males begin meiosis at puberty and complete meiosis rapidly after homolog desynapsis. In contrast, female meiosis is initiated during the fetal stage and arrests for a long time (e.g. up to 40 years in women) following homolog desynapsis (dictyate). This super‐long diplotene in females could allow oocytes to be competent to pass through MI upon oocyte meiotic resumption. Consistent with this idea, ZFP541/KCTD19 primarily functions at pachytene to regulate transcription and chromatin organization for proper meiotic progression. Moreover, several factors, such as SKP1, RPL10L, Eif4g3, MTAP2, Emi2, Cyclin A1, and MAPK, work at late prophase I and are required for male meiosis progression to metaphase I. However, they seem not to be required in female meiosis.^70^, ^71^, ^72^, ^73^, ^74^, ^75^, ^76^, ^77^, ^78^ There are also other possibilities that cannot be excluded, e.g. ZFP541/KCTD19 is a male‐specific regulator, or females have another factor with redundant roles.^39^

Another intriguing result in this study is that the absence of ZFP541/KCTD19 impairs the recruitment/localization of the sumoylation ligase RNF212 and ubiquitin ligase HEI10. RNF212 and HEI10 collaborate to selectively stabilize recombination intermediates to promote CO formation.^58^, ^59^, ^60^ The globally increased ubiquitination and sumoylation may disturb the recruitment/distribution of RNF212 and HEI10, which results in an increased number of RNF212 foci at mid and late pachytene but delayed recruitment of HEI10 at early/mid pachytene. Even at late pachytene, the mutants still have a reduced number of HEI10 foci than WT. Moreover, some HEI10 foci seem to be mislocalized on chromosomes since a fraction of nuclei only has HEI10 foci at/close to chromosome ends. Additionally, these aberrantly localized (and maybe a fraction of normally localized) HEI10 foci probably fail to recruit MLH1 given no nuclei with only chromosome ends localized MLH1 is observed, and pachytene nuclei have fewer MLH1 than HEI10 foci.

## 4 MATERIALS AND METHODS

### 4.1 Animals

Knockout mice for *Kctd19* (Gene ID: 279499) and *Zfp541* (Gene ID: 666528) under C57BL/6 background were constructed by injecting Cas9 mRNA and sgRNAs into zygotes as previously described^32^: sgRNA1 (TATAAAACTCGGGTG CTTTAAGG) and sgRNA2 (GTCCAGAGCAGTTAAGCCAATGG) to delete exons 2 and 3 of *Zfp541*, sgRNA3 (CGGGCTCCATGAGTCAGCAGAGG) and sgRNA4 (CTTGCTCTATGAGCAAG CCCTGG) to delete 163 of *Kctd19*. Knockout mice were confirmed by PCR of genomic DNA extracted from mouse toe tips. The PCR primers were P5 (CCACAGTCTTGTTGGATTGTTAAAG), P6 (ATTGGAGACATTCAGGTAGAGGAG), and P7 (CTGCCAATCAGATGAAAGGAGAAAAC) for the detection of DNA fragment deletion in *Zfp541*, and the PCR products were 599 bp for the wild‐type allele and 740 bp for the mutant allele. The PCR primers were P8 (ACTTCTTGTCAGGGTGCTTTC) and P9 (GATGCCCTTTTCTGGTGTCTC) for the detection of DNA fragment deletion in *Kctd19* and the PCR products were 566 bp for the wild‐type allele and 403 bp for the mutant allele. The homozygous knockout mice were obtained by mating heterozygous mice. The founder mice were backcrossed with wild‐type C57BL/6 mice for five generations before they were used in this study. The double mutant mice (*dKO*) were made by crossing *Kctd19* ^*+/−*^ with *Zfp541* ^*+/*−^ mice.

Mice used in this study were approved by the Animal Ethics Committee of the School of Medicine, Shandong University. All experiments in this study were reviewed and approved by the Animal Use Committee of the School of Medicine, Shandong University.

### 4.2 Antibodies

Rabbit polyclonal antibody against mouse ZFP541 amino acids (AA) 1‐104, Guinea pig polyclonal antibody against mouse ZFP541 AA 385‐556, Guinea pig polyclonal antibody against mouse KCTD19 AA 541‐760, and Rabbit polyclonal antibody against mouse TEX11 AA 788‐946 was prepared by Dai'an Biological Technology Incorporation; Guinea pig polyclonal antibody against mouse HEI10 AA 60‐276 was prepared by ABclonal Biotechnology co., Ltd. as previously described.^39^

Primary antibodies used for Western blot were as follows: Guinea pig anti‐KCTD19 (1:1000), rabbit anti‐ZFP541 (1:1000), mouse anti‐βACTIN (1:3000; Proteintech, 66009‐1‐Ig). Secondary antibodies used for Western blot were: HRP‐conjugated goat anti‐rabbit IgG (1:5000; Proteintech, SA00001‐2), HRP‐conjugated goat anti‐mouse IgG (1:5000; Proteintech, SA00001‐1), HRP‐conjugated affinipure goat anti‐guinea pig IgG (1:5000; Proteintech, SA00001‐12).

Primary antibodies used for immunostaining were as follows: mouse anti‐SYCP3 (1:500; Abcam, ab97672), rabbit anti‐SYCP3 (1:500; Abcam, ab15093), rabbit anti‐SYCP1 (1:500; Abcam, ab15090), guinea pig anti‐ZFP541 (1:100), mouse anti‐γH2AX (Ser139) (1:300; Millipore, 05‐636), mouse anti‐MLH1 (1:100; BD Biosciences, 550838), rabbit anti‐phospho‐histone H3 (Ser10) (1:200; Millipore, 06‐570), mouse anti‐TRF1 (1:200; Abcam, ab10579), human anti‐ACA (1:300; antibodies incorporated, 15‐234‐0001), telomere Probe (5 nM; PNA Bio, F1009), rabbit anti‐TRIP13 (1:200; Proteintech, 19602‐1‐AP), rabbit anti‐HORMAD1 (1:200; ABclonal, WG‐04467D), mouse anti‐CDK2 (1:100; Santa Cruz Biotechnology, SC‐6248), rabbit anti‐RPA2 (1:200; Abcam, ab76420), rabbit anti‐RAD51 (1:200; Thermo Fisher Scientific, PA5‐27195), rabbit anti‐DMC1 (1:200; Proteintech, 13714‐1‐AP50), rabbit anti‐TEX11 (1:200), rabbit anti‐MSH4 (1:200; Abcam, ab58666), rabbit anti‐RNF212 (1:200; a gift from Mengcheng Luo, Wuhan University), guinea pig anti‐HEI10 (1:150), guinea pig anti‐H1t (1:200), rabbit anti‐SUMO1 (1:200; Cell Signalling Technology, #4930), rabbit anti‐SUMO2/3 (1:200; Cell Signalling Technology, #4971), rabbit anti‐Ubiquitin (1:200; Ubiquigent, 68‐0121‐500), mouse anti‐20S proteasome β5(1:50; Santa Cruz A‐10 sc:393931), rabbit anti‐Histone H4 (1:200; Abcam, ab10807). Secondary antibodies were: CoraLite488‐conjugated goat anti‐mouse IgG (1:300; Proteintech, SA00013‐1), CoraLite488‐conjugated goat anti‐rabbit IgG (1:300; Proteintech, SA00013‐2), CoraLite594‐conjugated goat anti‐mouse IgG (1:300; Proteintech, SA00013‐3), CoraLite594‐conjugated goat anti‐rabbit IgG (1:300; Proteintech, SA00013‐4), Goat Anti‐Guinea pig IgG H&L (Alexa Fluor® 488) (1:300; Abcam,150185), Goat Anti‐Guinea pig IgG H&L (Alexa Fluor® 405) (1:300; Abcam,175678), Donkey Anti‐Mouse IgG H&L (Alexa Fluor® 405) (1:300; Abcam,175658), Donkey Anti‐Rabbit IgG H&L (Alexa Fluor® 405) (1:300; Abcam,175651).

### 4.3 Reverse transcriptional PCR (RT‐PCR)

Total RNA was isolated from different tissues of mice. The HiScript Q RT SuperMix for qPCR (+gDNA wiper) Kit (Vazyme Biotech Co., Ltd, R123‐01) was used to synthesize cDNA. To detect the *Kctd19/Zfp541* transcripts, PCR was performed using Taq Master Mix (Dye Plus) (Vazyme, P112‐03) and the following primers. Primers for *Zfp541*: P1 (on exon 5, GTTTCCATAGTGACCAGTG), P2 (on exon 6, GTAGGGAGAGCACTGAAA). Primers for *Kctd19*: P3 (on exon 5, GAATTTTCTACGCTCACACAAG), P4 (on exon 7, GTTCCCTGTGATGTATAGTCTG). Primers for the housekeeping gene β‐Actin: CATCCGTAAAGACCTCTATGCCAAC and ATGGAGCCACCGATCCACA. Primers for *Zfp541* (Exon 2‐3) knockout efficiency: P10 (on exon 1, ATGGAGCCATACAGTCTTGGG) and P11 (on exon 14, TCACCACTGCAAGGGGCC). Primers for *Kctd19* knockout efficiency: P12 (on exon 1, TCACGAGTCTGTCGTCT) and P13 (on exon 4, CAGTAGTGCACTTCCTCTTCT). PCR products were analysed on 1.5% agarose gels or by DNA sequencing.

### 4.4 Co‐immunoprecipitation

Testes were homogenized in lysis buffer (120 mM NaCl, 50 mM Tris–HCl, pH 8.0, 20 mM NaF, 20 mM β‐glycerophosphate, 6 mM EGTA, pH 8.0, 1 mM EDTA, 1 mM DTT, 1% NP‐40, 1× protease inhibitor cocktail) for 30 min on ice, then centrifuged at 12000*g* for 20 min at 4°C. The supernatant and co‐immunoprecipitation antibody were incubated on a rotary mixer overnight at 4°C. The next day, protein A/G magnetic beads (MCE, HY‐K0202) were mixed with a supernatant‐antibody complex and incubated for 2 h. Using the lysis buffer wash the magnetic beads three times and discard the supernatant. Add 1x SDS loading buffer to resuspend beads and boil at 95°C for 5 min. Proteins were separated on 10% SDS‐PAGE gels, transferred onto immobilon‐NC transfer membranes (Millipore, HATF00010), and immunoblotted with indicated primary and secondary antibodies.

### 4.5 Yeast two‐hybrid assay

The *Kctd19* long or short variant cDNA was cloned into the pGBKT7 plasmid as baits. *Zfp541* cDNAs were cloned into the pGADT7 vector as preys. Yeast two‐hybrid assays (Y2H) were performed as described previously.^39^ The bait and prey plasmids were co‐transformed into the Y2H gold strain. The interaction between bait and prey was detected on SD‐ Trp‐Leu‐His plates.

### 4.6 Mouse fertility test

The fertility test was performed in 8‐week male and female mice with indicated genotypes. Each heterozygous male mouse was mated with one heterozygous or one knockout female mouse. Each knockout male mouse was mated with one heterozygous female mouse. Vaginal plugs were checked every 3 days. Pregnancies and pups were recorded. The fertility test lasted for 6 months.

### 4.7 Histological analysis

Cauda epididymis and testes from adult mice were fixed in Bouin's solution (Sigma, HT10132‐1L) at 4°C for 24 h, then embedded in paraffin, and cross‐sectioned. Sections mounted on glass slides were dewaxed and stained with haematoxylin and eosin (HE) for cauda epididymis, or with Periodic acid Schiff (PAS) for testes using PAS staining kit (KeyGEN BioTECH, KGA222). For in situ cell death analysis, testes from adult mice were fixed in 4% Paraformaldehyde Fix Solution (Beyotime, P0099), and TUNEL staining was performed using an In Situ Cell Death Detection Kit, POD (Roche, 11684817910) and DAB Substrate kit (Solarbio, DA1010).

### 4.8 Chromosome spreading and immunostaining

The seminiferous tubules from decapsulated testes were treated in hypotonic buffer (30 mM Tris, 17 mM trisodium citrate dihydrate, 50 mM sucrose, 5 mM EDTA, 0.5 mM PMSF, and 0.5 mM DTT, pH 8.2) at room temperature for 30 min. The fragments of seminiferous tubules were moved to 0.1 M sucrose and torn with sharp tweezers to release spermatocytes. The cell suspension was then spread onto glass slides pretreated with 1% PFA containing 0.15% Triton X‐100.^79^ The slides were dried at room temperature and used for subsequent immunostaining.

For immunostaining, slides were dipped in 0.4% Photo‐flo for 30 s and then incubated in 1× PBS for 5 min. Slides were blocked with 5% BSA for 30 min, then incubated with primary antibodies at 4°C overnight. After being washed with 1× PBS three times, slides were incubated with secondary antibodies at room temperature for 1 h and mounted using the mounting medium (with/without DAPI) (Solarbio, S2110/S2100).

### 4.9 Chromosome spreading for Giemsa staining

Chromosome spreading for Giemsa staining was performed as previously reported with minor modifications.^71^ Briefly, testes freshly isolated from 10‐week mice were decapsulated and rinsed gently in 2.2% (w/v) trisodium citrate dihydrate, torn with tweezers, and gently pipetted 3–5 times. The suspension was passed through a 40 μm cell strainer. Cells in the filtrate were collected by centrifugation, then resuspended in 1% (w/v) trisodium citrate dehydrate at 37°C, and incubated at 37°C for 13 min. After centrifugation, cells were resuspended and fixed in Carnoy's solution at room temperature for 30 min. This step was repeated once. The supernatant was removed and single cells were resuspended in residual Carnoy's solution. For chromosome spreading, the cell suspension was dropped onto cold slides pretreated at −20°C. The slides were dried at room temperature and stained with Giemsa stain (KeyGEN BioTECH, KGA228, diluted in ddH_2_O 1:10) according to the manufacturer's instructions.

### 4.10 Culture of mouse testicular cells and okadaic acid (OA) treatment

Mouse testicular cells were cultured in vitro as reported.^70^, ^80^ Briefly, testes freshly isolated from 8‐week mice were decapsulated and rinsed gently in 1× Krebs buffer (120 mM NaCl, 4.8 mM KCl, 25.2 mM NaHCO_3_, 1.2 mM KH_2_PO_4_, 1.2 mM MgSO_4_, 1.3 mM CaCl_2_, and 11.1 mM dextrose) for several times. The testis seminiferous tubules were digested in 1× Krebs buffer with collagenase I (1.6 mg/mL) at 33°C for 10 min with shaking. Then, the seminiferous tubules were transferred into 1× Krebs buffer with 1.6 mg/mL collagenase I, 0.01% trypsin, and a proper amount of DNase I, and treated at 33°C for 6 min with gentle shaking. The cell suspension was passed through a 100 μm nylon mesh. ~1 − 4 × 10^6^ testicular cells were suspended in 1 mL MEMα with 7.5% penicillin, 5% streptomycin, 0.59% HEPES, 0.29% DL‐lactic acid sodium salt, and 5% FBS, then were seeded into 6‐well plate and cultured overnight at 32°C. The next morning, a few cells were used to examine cell viability, and the rest was treated with OA (Cell Signalling Technology, 5934S) at a final concentration of 4 μM. After 5 h, cells were harvested for chromosome spreading and Giemsa staining.

### 4.11 Fluorescence in situ hybridization

Slides containing spermatocytes were dipped in 0.4% Photo‐flo for 30 s three times and then incubated 1× PBS for 5 min. Dehydrate the slides with 70%, 85%, and 100% ethanol, respectively. The slides were heated on a metal bath at 80°C for 5 min. The telomeric probe (PNA Bio, F1009) was diluted by hybridization buffer (20 mM Na_2_HPO_4_, 20 mM Tris, 60% Formamide, 30 mM Sodium citrate, 300 mM NaCl, 0.1 μg/mL salmon sperm DNA, pH 7.4) to 400 nM and used as working concentration. The slides incubated with a telomere probe were put on a metal bath at 85°C 10 min for DNA denaturing and then transfer to a wet box at 4°C for 22–24 h. The slides were washed with the washing solution (30 mM Sodium citrate, 300 mM NaCl, 0.1% Tween‐20) twice in the 60°C hybrid furnace. After blocked with 5% BSA for 30 min, they were then incubated with primary/secondary antibodies. Finally, the slides were mounted with a mounting medium with DAPI.

### 4.12 Quantitative reverse transcription PCR (RT‐qPCR)

The RNA was extracted by RNAsimple Total RNA Kit (TIANGEN, DP419) and reverse transcribed into cDNA. Real‐time fluorescent quantitative PCR was performed by using 2× Universal SYBR Green Fast qPCR Mix (ABclonal, RK21203) and detected by Roche LightCycler 480 detection system. Primers were listed in Table S1. Each experiment was performed for three replicates. Actin was used as an internal control and gene expression levels were calculated using 2^−△△Ct^.

### 4.13 Imaging

The immunostained slides were visualized and imaged using an epifluorescence microscope (BX52, Olympus) or a rotary laser confocal microscope (Dragonfly, Andor Technology), driven by Fusion Software.

### 4.14 RNA‐seq

Testicular tissue from 8‐week mice was digested into single cells using hyaluronidase (Sigma, H3506), collagenase (Worthington Biochemical Corporation, LS004194), 0.25% trypsin(KeyGEN BioTECH, KGA25200) and DNaseI(Solarbio, D8071). Pachytene spermatocytes were isolated after sedimentation by 2%–4% BSA. Total RNA was extracted using TRIzol reagent (CWBIO, CW0580) according to the manufacturer's instructions. Total amounts and integrity of RNA were assessed using the RNA Nano 6000 Assay Kit of the Bioanalyzer 2100 system (Agilent Technologies, USA). Library DNAs were prepared using NEBNext® Ultra™ RNA Library Prep Kit and sequenced using Illumina NovaSeq 6000 with 150 bp pair‐end reads by Novogene Co., Ltd. Differential expression analysis was performed using the DESeq2 R package (1.20.0). GTF file was derived from UCSC mm10. *p*‐value < 0.05 and |log2(foldchange)|>1 were set as the threshold for significantly differential expression. Gene Ontology analysis of differentially expressed genes was performed using Metascape (http://metascape.org).

### 4.15 ATAC‐seq

8‐week mice pachytene spermatocytes were obtained as those for RNA‐seq. DNA accessibility is probed by the hyperactive Tn5 transposase, which inserts sequencing adapters into accessible regions of chromatin. Sequencing reads were then used to infer regions of increased accessibility. ATAC‐seq libraries were sequenced using the DNBSEQ platform in PE50 mode by Shenzhen Huada Gene Technology Co., Ltd.

### 4.16 Statistical analysis

Statistical analyses were conducted using Graph‐Pad PRISM version 8.0.2. The error bars were shown as mean ± SEM, SD, or 95% confidence interval as indicated in figure legends. The statistical significance of the differences between the two sets of data was measured by Student's *t*‐test or Chi‐square test as indicated. Sample sizes were described in figure legends. The levels of significance were indicated as *p* ≥ 0.05 (n.s. not significant); *p* < 0.05 (*); *p* < 0.01 (**); or *p* < 0.001 (***).

## AUTHOR CONTRIBUTIONS

Xu Zhou, Liangran Zhang and Shunxin Wang conceived the study and designed experiments. Charlie Degui Chen and Kailun Fang provided the knockout mice. Xu Zhou, Yanlei Liu, Weidong Li, Yingjin Tan, Jiaming Zhang, Xiaoxia Yu, Guoqiang Wang, Yanan Zhang, and Yongliang Shang did experiments. Xu Zhou, Liangran Zhang and Shunxin Wang analysed the data and wrote the manuscript with inputs and edits from all authors.

## FUNDING INFORMATION

This work was supported by The National Natural Science Foundation of China (32070837, 32225015); National Key Research & Development Program of China (2021YFC2700103; 2022YFC2702602); Taishan Scholars Youth Project of Shandong Province.

## CONFLICT OF INTEREST STATEMENT

The authors declare no competing interests.

## Supporting information


**Data S1:** Supporting Information.

## Data Availability

The data for RNA‐seq and ATAC‐seq were deposited at NCBI under Bioproject# PRJNA980436. ZFP541 ChIP‐seq data were downloaded from NCBI under Bioproject# PRJNA722264. Additional information required to reanalyse the data reported in this paper is available from the lead contact upon reasonable request.
