# Supplementary material for: ZFP541 and KCTD19 regulate chromatin organization and transcription programs for male meiotic progression
Source: Cell Prolif. 2023 Nov 3;57(4):e13567. doi: 10.1111/cpr.13567 (PMC10984108; doi:10.1111/cpr.13567)
Supplement: Supplementary file 1 — Data S1: Supporting Information. [file CPR-57-e13567-s001.pdf]

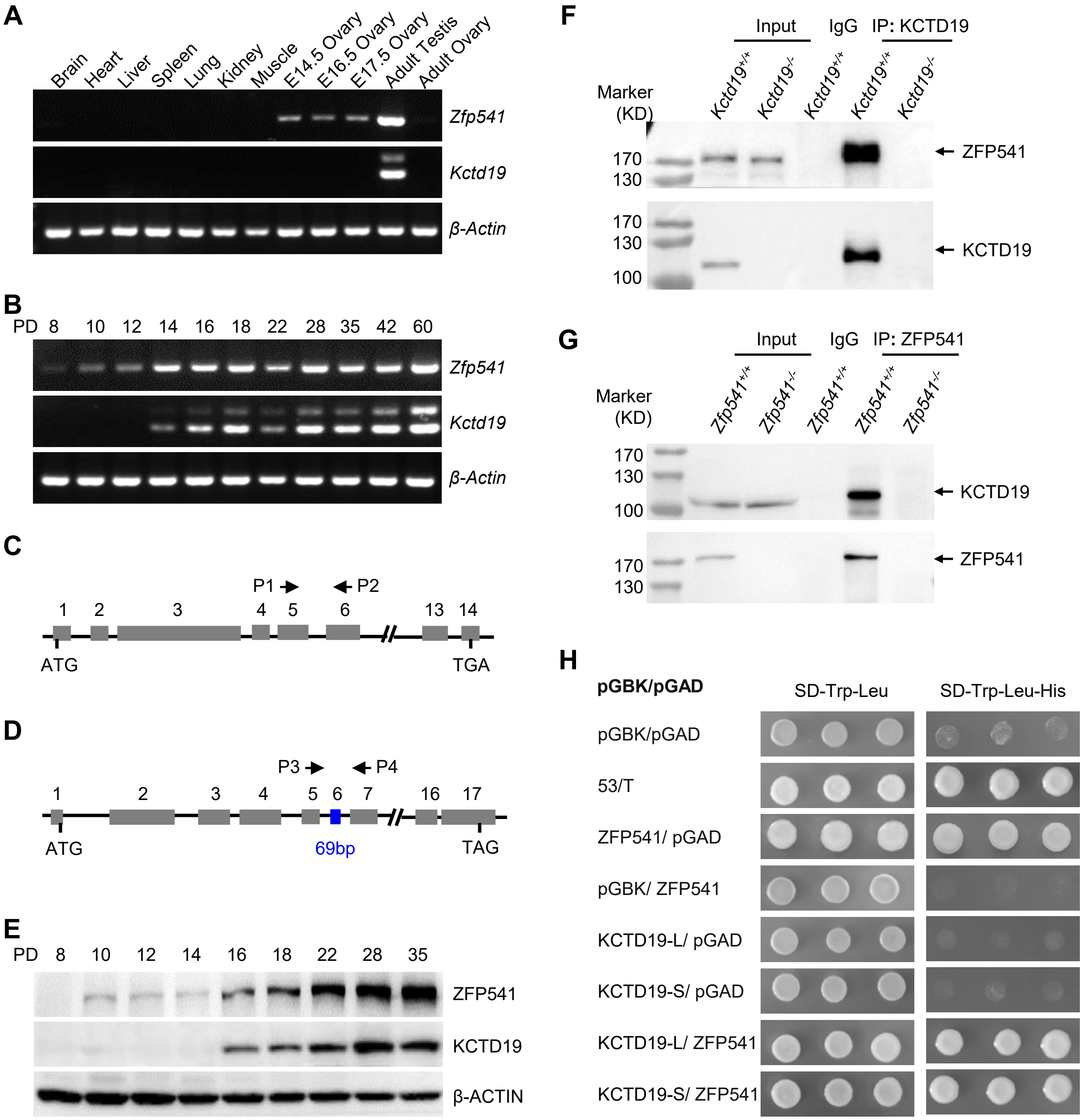


**Figure S1. ZFP541 and KCTD19 preferentially express during spermatogenesis and interacts with each other. (A, B)** RT-qPCR to show the expression of *Zfp541* and *Kctd19* in different tissues (A) and postnatal testis (B). Note, the two bands (variants) for *Kctd19*. **(C, D)** The schematic diagram to show the gene structures of *Zfp541* (C) and *Kctd19* (D). The positions of primer pair P1 and P2 for the detection of *Zfp541* transcripts, and P3 and P4 for the detection of *Kctd19* transcripts, are indicated. Exon 6 is absent in the short transcript of *Kctd19*. **(E)** Western blot to show ZFP541 and KCTD19 proteins in postnatal testis. (**F, G**) Co-immunoprecipitation to show the interaction between ZFP541 and KCTD19 in testis lysates. **(H)** Yeast two-hybrid experiments to examine the interaction between ZFP541 and KCTD19. pGBK-p53/pGAD-T is a positive control. pGBK-ZFP541 shows self-activation. KCTD19-L, the long isoform of KCTD19; KCTD19-S, the short isoform of KCTD19.

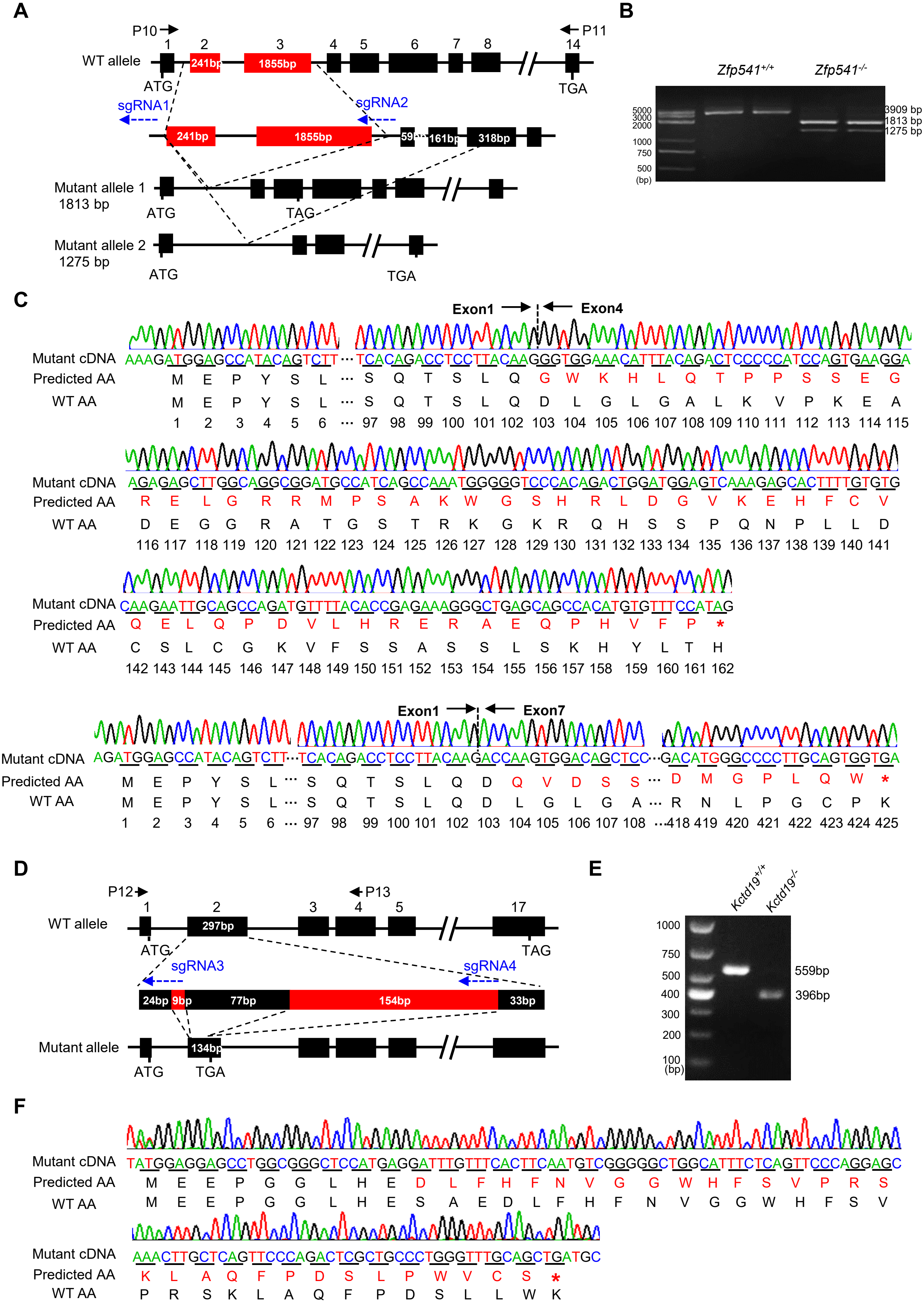
A
P10
P11
1
2
3
4
5
6
7
8
14
WT allele
241bp
1855bp
ATG
TGA
sgRNA1
sgRNA2
241bp
1855bp
161bp
318bp
Mutant allele 1
1813 bp
ATG
TAG
Mutant allele 2
1275 bp
ATG
TGA
B
Zfp541+/+
Zfp541-/-
5000
3000
2000
1000
750
500
(bp)
3909 bp
1813 bp
1275 bp
C
Exon1
Exon4
Mutant cDNA
Predicted AA
WT AA
Exon1
Exon7
D
P12
P13
1
2
3
4
5
17
WT allele
297bp
ATG
TAG
sgRNA3
sgRNA4
24bp
9bp
77bp
154bp
33bp
Mutant allele
134bp
ATG
TGA
E
Kctd19+/+
Kctd19-/-
1000
750
500
400
300
200
100
(bp)
559bp
396bp
F
Mutant cDNA
Predicted AA
WT AA

**Figure S2. The knockout of *Zfp541* and *Kctd19* is confirmed by RT-PCR and subsequent sequencing.** **(A)** The schematic diagram of the *Zfp541* knockout allele. Exon 2 and Exon 3 are deleted. **(B)** Two short transcripts are observed in the mutant by RT-PCR using template cDNA from 8-week testes and primers P10 and P11. **(C)** The DNA sequences of RT-PCR products from (B) and the predicted amino acids. The two transcripts have frameshift from the 103rd and 104th amino acids, respectively. The new stop codon (TAG/TGA) is indicated by a star (*). **(D)** The schematic diagram of the *Kctd19* knockout allele. Two fragments within exon 2 are deleted. **(E)** A short transcript is observed in the mutant by RT-PCR using template cDNA from 8-week testes and primers P12 and P13. **(F)** The DNA sequences of RT-PCR product from (E) and the predicted amino acids. The frameshift from the 10th amino acid and the new stop codon (TGA; indicated by a star *) in the mutant allele are indicated.

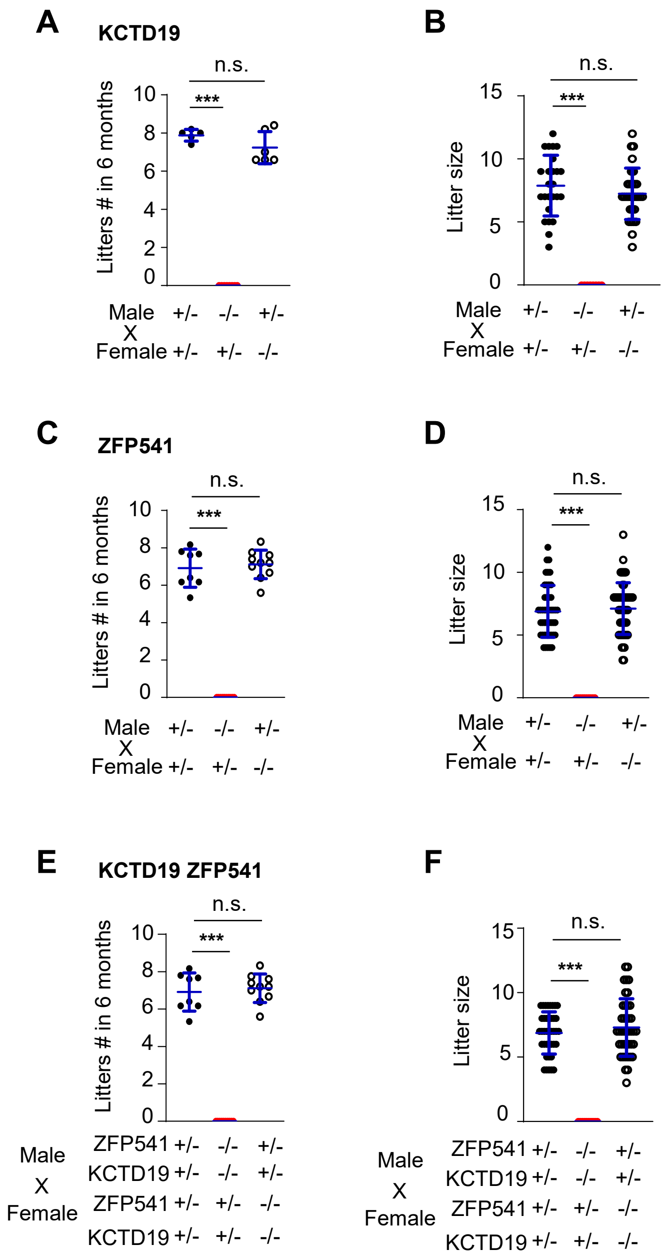


**Figure S3. Both ZFP541 and KCTD19 are required for male fertility.**

Each heterozygous male mouse was bred to one heterozygous or one knockout female. Each knockout male mouse was bred to one heterozygous female. All females were plugged. **(A, B)** *Kctd19*$^{-/-}$ male mice are sterile, while *Kctd19*$^{-/-}$ female mice show normal fertility. n = 5, 8, and 6 breeding pairs from left to right, respectively. **(C, D)** *Zfp541*$^{-/-}$ male mice are sterile, while *Zfp541*$^{-/-}$ female mice show normal fertility. n = 8, 10, and 10 breeding pairs from left to right, respectively. **(E, F)** *Kctd19*$^{-/-}$*Zfp541*$^{-/-}$ male mice are sterile, while *Kctd19*$^{-/-}$ *Zfp541*$^{-/-}$ female mice show normal fertility. n = 8, 10, and 8 breeding pairs from left to right, respectively. Error bar, mean ± SD. n.s. (not significant), $p \geqslant 0.05$; ***, $p<0.001$; two-tailed Student's t-test.

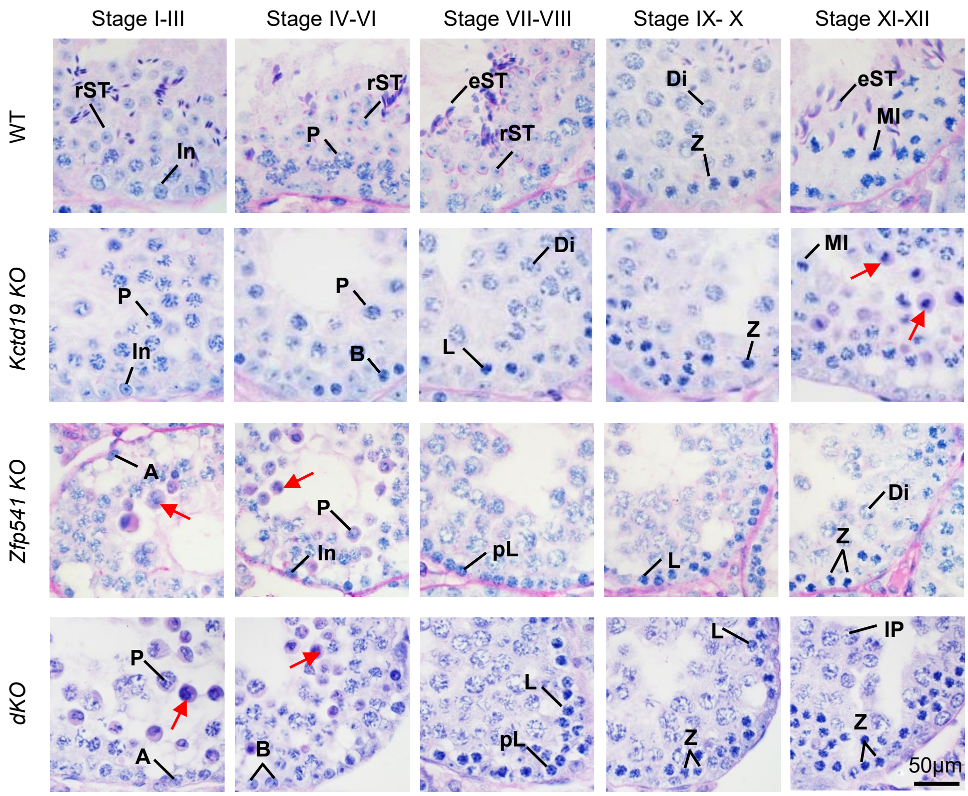


**Figure S4. Apoptotic spermatocytes in mutant male mice.**

Representative images of Periodic acid Schiff (PAS) staining to show the various stages of the cycle of the seminiferous epithelium in 8-week mice. A considerable number of apoptotic metaphase I-like (aMI-like) spermatocytes are observed in *Kctd19*$^{-/-}$ testes. A large number of apoptotic pachytene spermatocytes are observed in *Zfp541*$^{-/-}$ and *Kctd19*$^{-/-}$ *Zfp541*$^{-/-}$ testes. Red arrows, apoptotic spermatocytes. A, A spermatogonia; B, B spermatogonia; In, intermediate spermatogonia; L, leptotene; Z, zygotene; P, pachytene; Di, diplotene; MI, metaphase I; rST, round spermatid; eST, elongated spermatid.

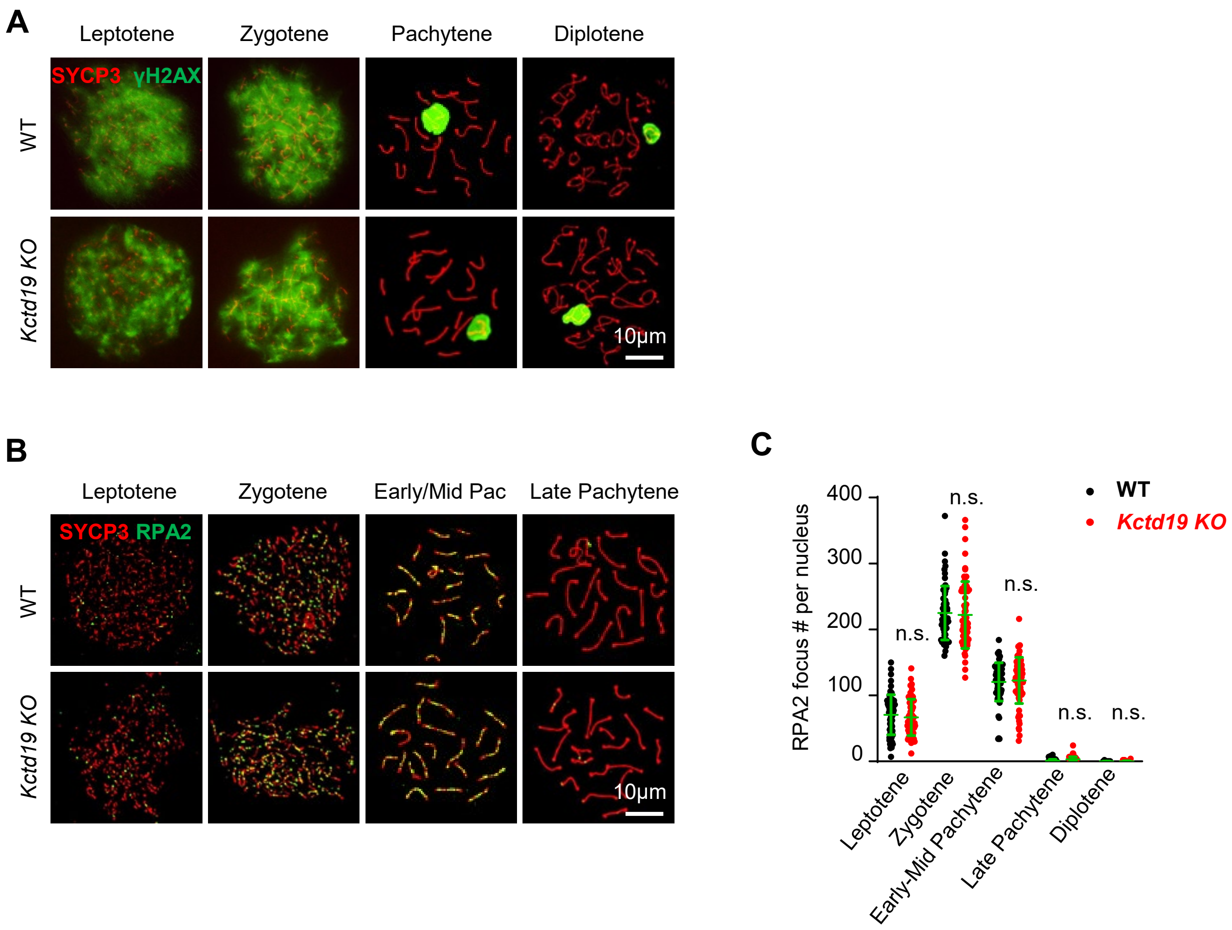


**Figure S5. *Kctd19*$^{-/-}$ mice do not affect DSB repair.**

**(A)** γH2AX appears normal in *Kctd19*$^{-/-}$ spermatocytes. Representative images to show γH2AX in spermatocytes at different stages from 8-week WT and *Kctd19 KO* mice. **(B)** Representative images to show RPA2 foci on spermatocyte chromosomes from 8-week mice by co-immunostaining of RPA2 and SYCP3 antibodies. **(C)** Quantification of RPA2 focus number at different stages. Data from three WT and three *Kctd19*$^{-/-}$ mice were combined, respectively, since no difference is observed between different mice with the same genotype. From left to right, n =71, 60, 71, 80, and 76 nuclei from three WT mice and n=67, 63, 70, 72, and 83 nuclei from three *Kctd19*$^{-/-}$ mice. Error bar, mean ± SD; n.s. (not significant), $p \geqslant 0.05$; two-tailed Student's t-test.

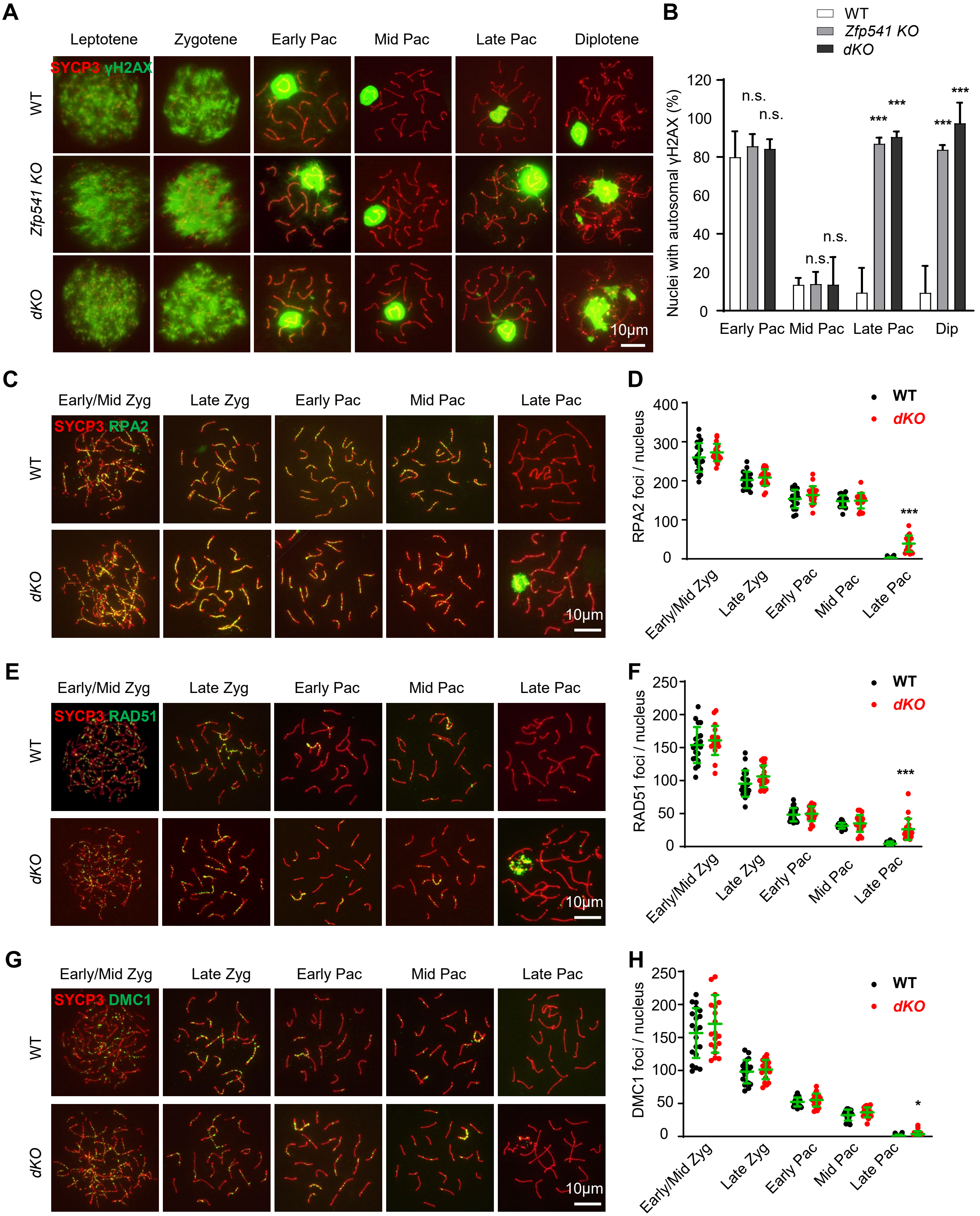

A
Leptotene
Zygotene
Early Pac
Mid Pac
Late Pac
Diplotene
SYCP3 γH2AX
WT
Zfp541 KO
dKO
10μm
B
WT
Zfp541 KO
dKO
Nuclei with autosomal γH2AX (%)
n.s.
***
Early Pac
Mid Pac
Late Pac
Dip
C
Early/Mid Zyg
Late Zyg
SYCP3 RPA2
D
RPA2 foci / nucleus
E
SYCP3 RAD51
F
RAD51 foci / nucleus
G
SYCP3 DMC1
H
DMC1 foci / nucleus
*

**Figure S6. *Zfp541$^{-/-}$ Kctd19$^{-/-}$* spermatocytes show recurrent DSBs.**

**(A)** Representative images to show γH2AX in spermatocytes at different stages. **(B)** Quantification of nuclei with γH2AX on autosomes at pachytene and diplotene. n=163, 106, 113, 134, 52, 68, 86, 140, 149, 97, 86, 58 spermatocytes from left to right. Error bar, 95% confidence interval. n.s. (not significant), $p \geqslant 0.05$; ***, $p<0.001$; Chi-square test. **(C, D)** Representative images and quantification to show RPA foci in spermatocytes at different stages. n=20 for each genotype at each stage. Error bar, mean ± SD. ***, $p<0.001$; two-tailed Student's t-test. **(E, F)** Representative images and quantification to show RAD51 foci in spermatocytes at different stages. n=20 for each genotype at each stage. Error bar, mean ± SD. ***, $p<0.001$; two-tailed Student's t-test. **(G, H)** Representative images and quantification to show DMC1 foci in spermatocytes at different stages. n=20 for each genotype at each stage. Error bar, mean ± SD. *, $p<0.05$; two-tailed Student's t-test. Note: (1) 32 of 56 (57.14%) late pachytene spermatocytes show a big patch of RPA around XY chromosomes, 25 of these 32 (78.125%) spermatocytes have detached chromosome ends, and 11 of the 24 (45.83%) spermatocytes without patches of RPA have detached chromosome ends; (2) 14 of 42 (33.33%) late pachytene spermatocytes show a big patch of RAD51 around XY chromosomes, 13 of these 14 (92.86%) spermatocytes also have detached chromosome ends, and 15 of the 28 (53.57%) spermatocytes without patches of RAD51 has detached chromosome ends.

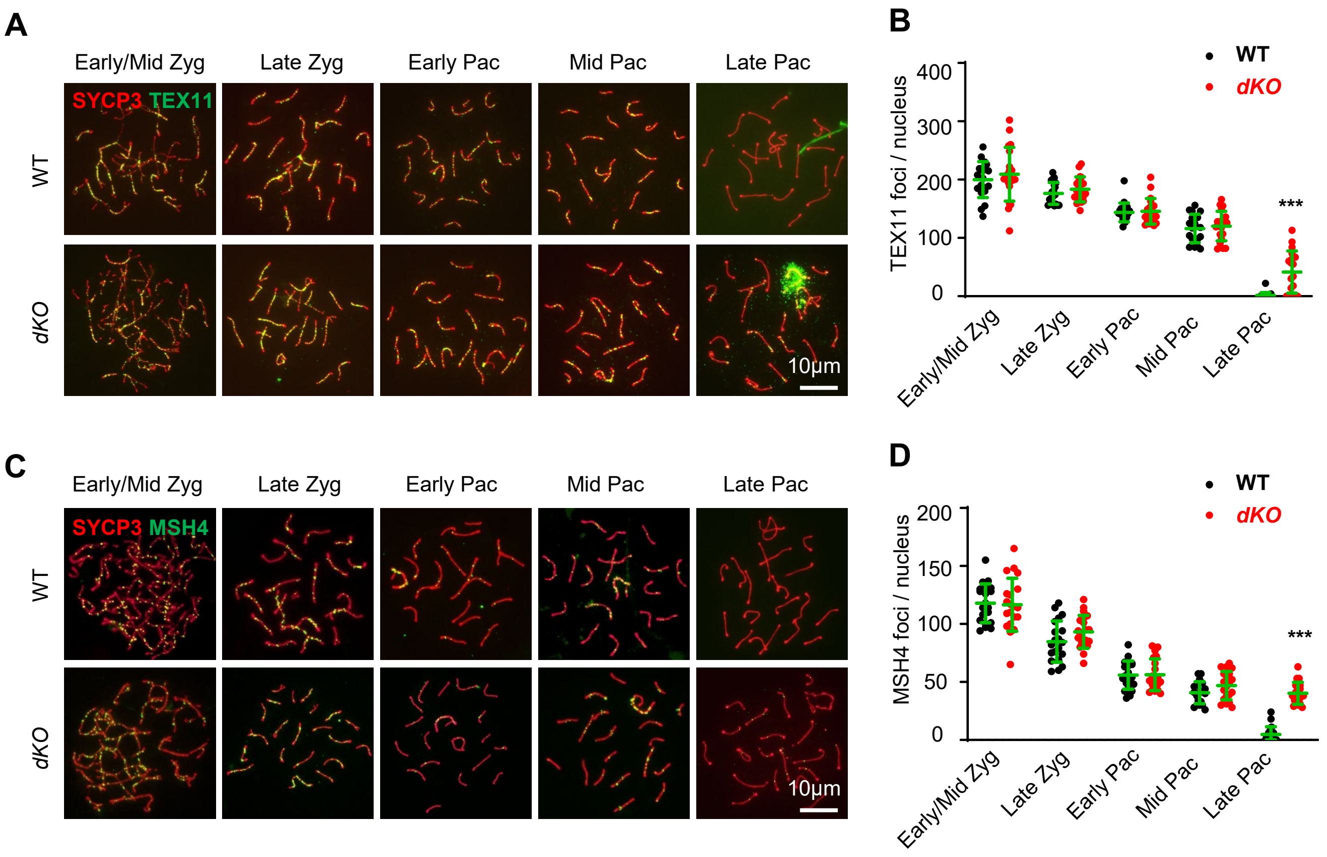


**Figure S7. Examination of TEX11 and MSH4 in *Zfp541$^{-/-}$ Kctd19$^{-/-}$* spermatocytes.**

**(A, B)** Representative images and quantification to show TEX11 foci in spermatocytes at different stages. n=20 for each genotype at each stage. Error bar, mean ± SD; n.s. (not significant), $p \geqslant 0.05$; ***, $p<0.001$; two-tailed student's t-test. **(C, D)** Representative images and quantification to show MSH4 foci in spermatocytes at different stages. n=20 for each genotype at each stage. Error bar, mean ± SD; n.s. (not significant), $p \geqslant 0.05$; ***, $p<0.001$; two-tailed student's t-test.

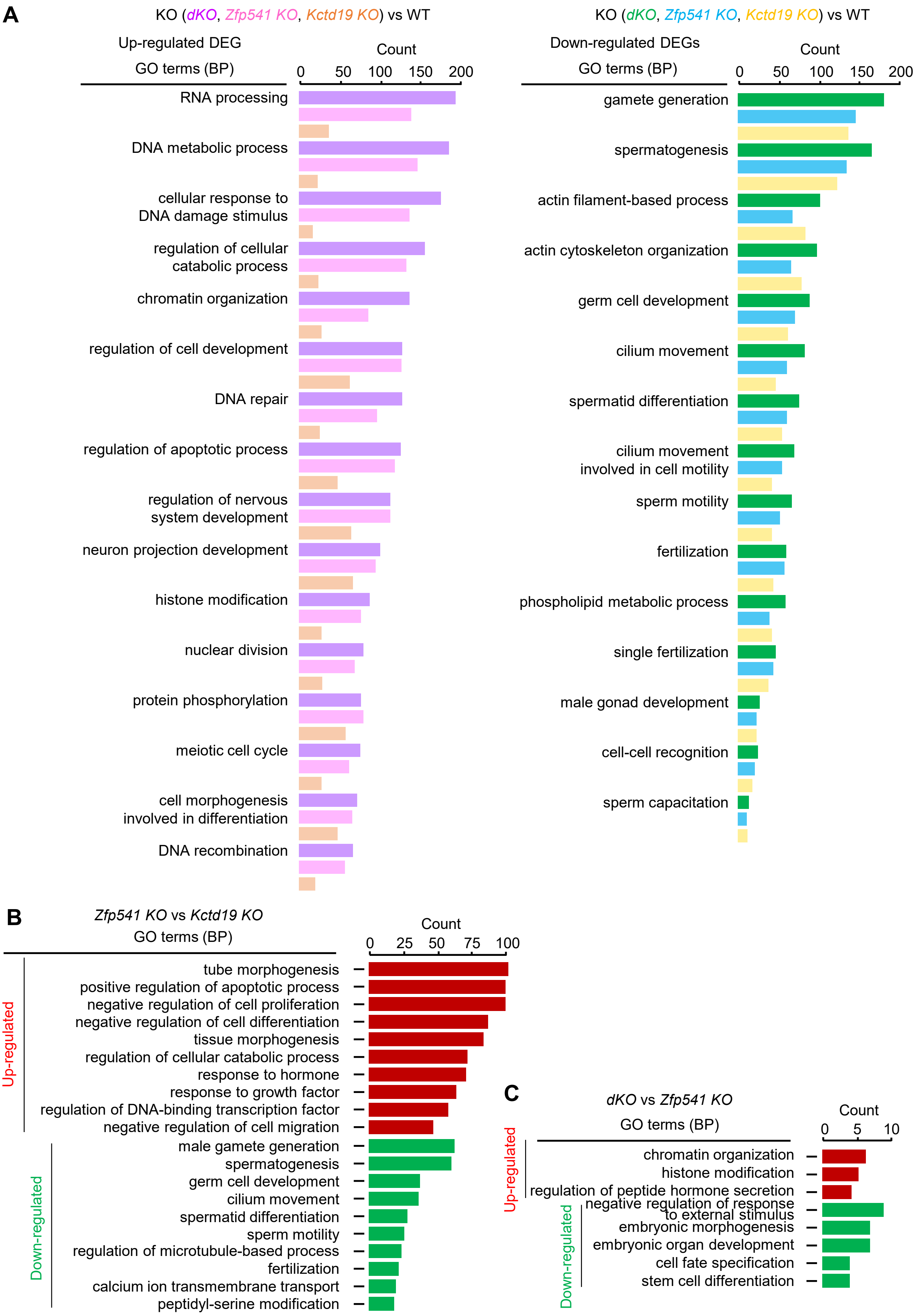
A
KO (dKO, Zfp541 KO, Kctd19 KO) vs WT
Up-regulated DEG
GO terms (BP)
Count
0
50
100
150
200
RNA processing
DNA metabolic process
cellular response to DNA damage stimulus
regulation of cellular catabolic process
chromatin organization
regulation of cell development
DNA repair
regulation of apoptotic process
regulation of nervous system development
neuron projection development
histone modification
nuclear division
protein phosphorylation
meiotic cell cycle
cell morphogenesis involved in differentiation
DNA recombination
KO (dKO, Zfp541 KO, Kctd19 KO) vs WT
Down-regulated DEGs
GO terms (BP)
Count
0
50
100
150
200
gamete generation
spermatogenesis
actin filament-based process
actin cytoskeleton organization
germ cell development
cilium movement
spermatid differentiation
cilium movement involved in cell motility
sperm motility
fertilization
phospholipid metabolic process
single fertilization
male gonad development
cell-cell recognition
sperm capacitation
B
Zfp541 KO vs Kctd19 KO
GO terms (BP)
Count
0
25
50
75
100
Up-regulated
tube morphogenesis
positive regulation of apoptotic process
negative regulation of cell proliferation
negative regulation of cell differentiation
tissue morphogenesis
regulation of cellular catabolic process
response to hormone
response to growth factor
regulation of DNA-binding transcription factor
negative regulation of cell migration
Down-regulated
male gamete generation
spermatogenesis
germ cell development
cilium movement
spermatid differentiation
sperm motility
regulation of microtubule-based process
fertilization
calcium ion transmembrane transport
peptidyl-serine modification
C
dKO vs Zfp541 KO
GO terms (BP)
Count
0
5
10
Up-regulated
chromatin organization
histone modification
regulation of peptide hormone secretion
Down-regulated
negative regulation of response to external stimulus
embryonic morphogenesis
embryonic organ development
cell fate specification
stem cell differentiation

**Figure S8. GO enrichment analysis of DEGs.**

**(A)** GO enrichment analysis of DEGs between each mutant and WT. **(B)** GO enrichment analysis of DEGs between *Zfp541 KO* and *Kctd19 KO*. **(C)** GO enrichment analysis of DEGs between *dKO* and *Zfp541 KO.*

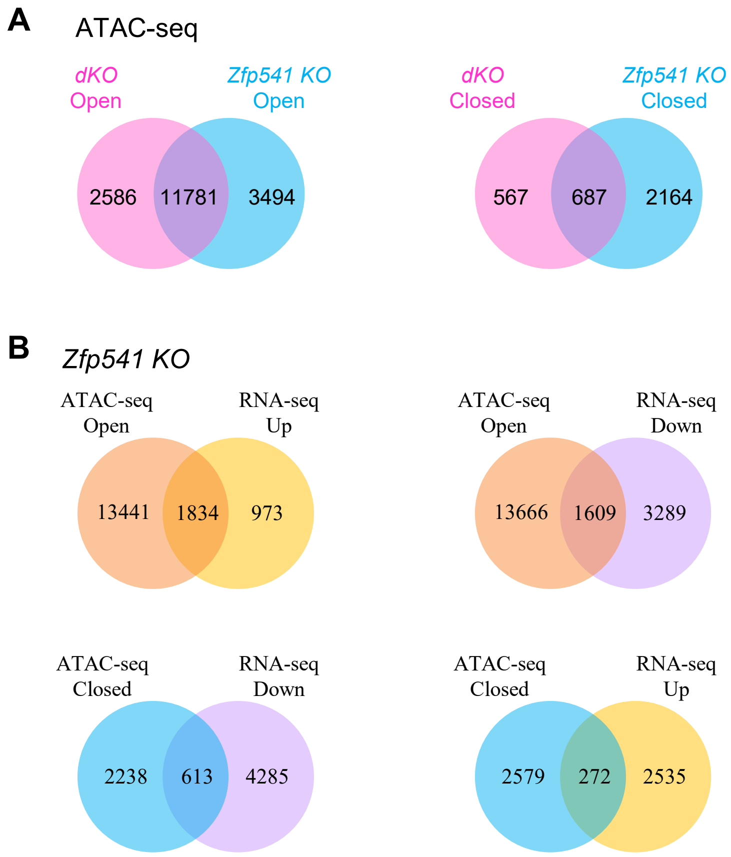


**Figure S9. Analysis of DEGs associated with altered chromatin accessibility.**

**(A)** Venn plots to show the numbers of overlapped regions with increased and decreased chromatin accessibility between *Zfp541 KO* and *dKO* spermatocytes, respectively. **(B)** Venn plots to show the numbers of overlapped regions with altered chromatin accessibility and DEGs from RNA-seq in *Zfp541 KO*.

**A**

Contribution of each TF to DEGs

| Up-regulated TF (17) | Tcf3 | Tcf12 | Smad4 | Smad2 | Usf2 | Ascl1 | Ptf1a | Mef2a | Egr1 | Sox3 | Tbr1 | Sox4 | Elk4 | Nr2f2 | Barx1 | Atf1 | Pbx3 |
|---|---|---|---|---|---|---|---|---|---|---|---|---|---|---|---|---|---|
| DEG% | 44.09 | 41.12 | 38.81 | 38.46 | 30.99 | 21.38 | 17.48 | 13.96 | 9.71 | 8.67 | 8.38 | 5.22 | 1.65 | 0.44 | 0.01 | 0.00 | 0.00 |

| Down-regulated TF (8) | Klf4 | Srf | Esrrb | Six2 | Lhx2 | Foxa3 | Bcl11a | Klf9 |
|---|---|---|---|---|---|---|---|---|
| DEG% | 47.32 | 33.39 | 22.80 | 15.42 | 13.78 | 0.79 | 0.00 | 0.00 |

**B**

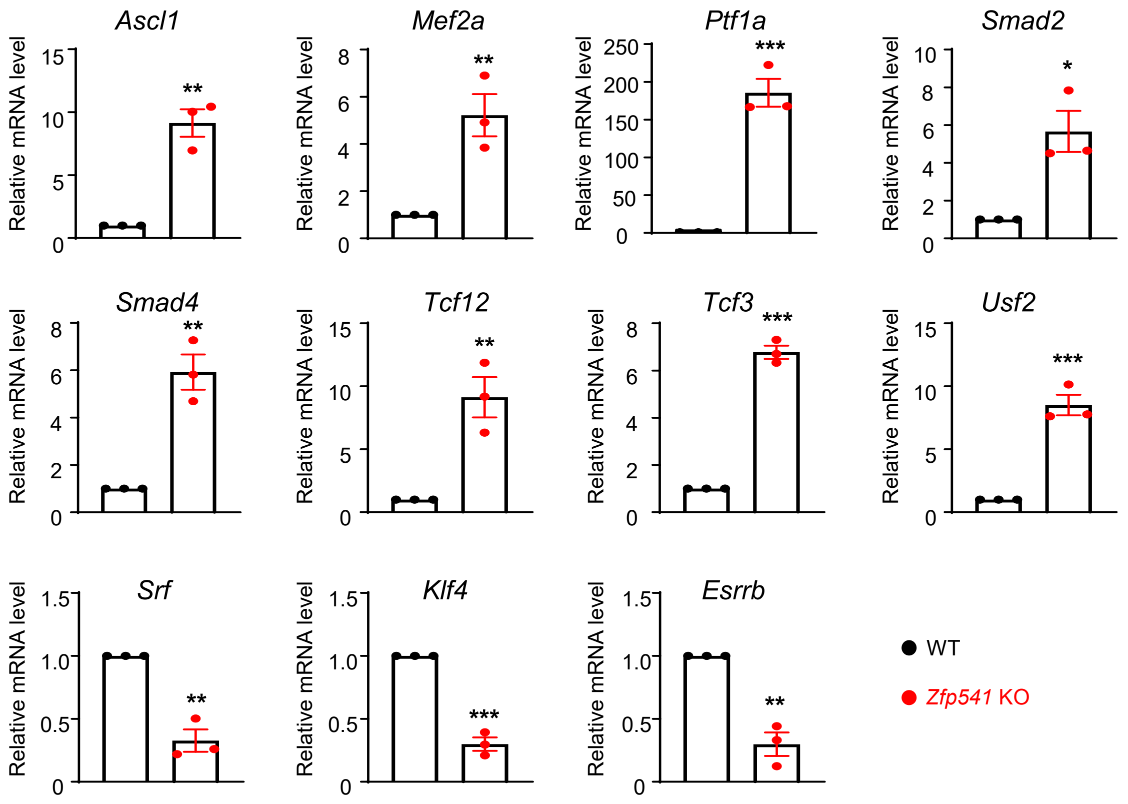


**Figure S10. Identified TFs and their contribution to DEGs.**

**(A)** The contribution of each candidate TF to DEGs. **(B)** RT-qPCR to validate the altered expression of selected TFs. The mRNA was extracted from WT and *Zfp541* KO pachytene spermatocytes and the expression of selected TFs was examined by RT-qPCR. Error bar, mean ± SEM (n=3). *, *p*<0.05; **, *p*<0.01; ***, *p*<0.001; two-tailed Student's t-test.

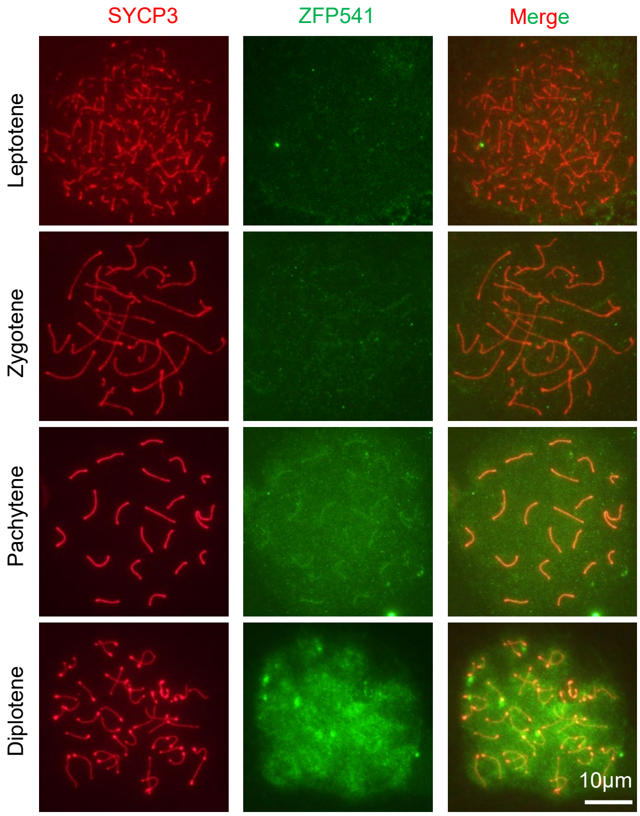


**Figure S11. Immunostaining of ZFP541 in spread spermatocytes.**

SYCP3 staining (red) of the chromosome axis is used to indicate the stages of meiotic prophase I. ZFP541 (green) is undetectable in leptotene, barely observed at zygotene, and easily observed at pachytene and diplotene.

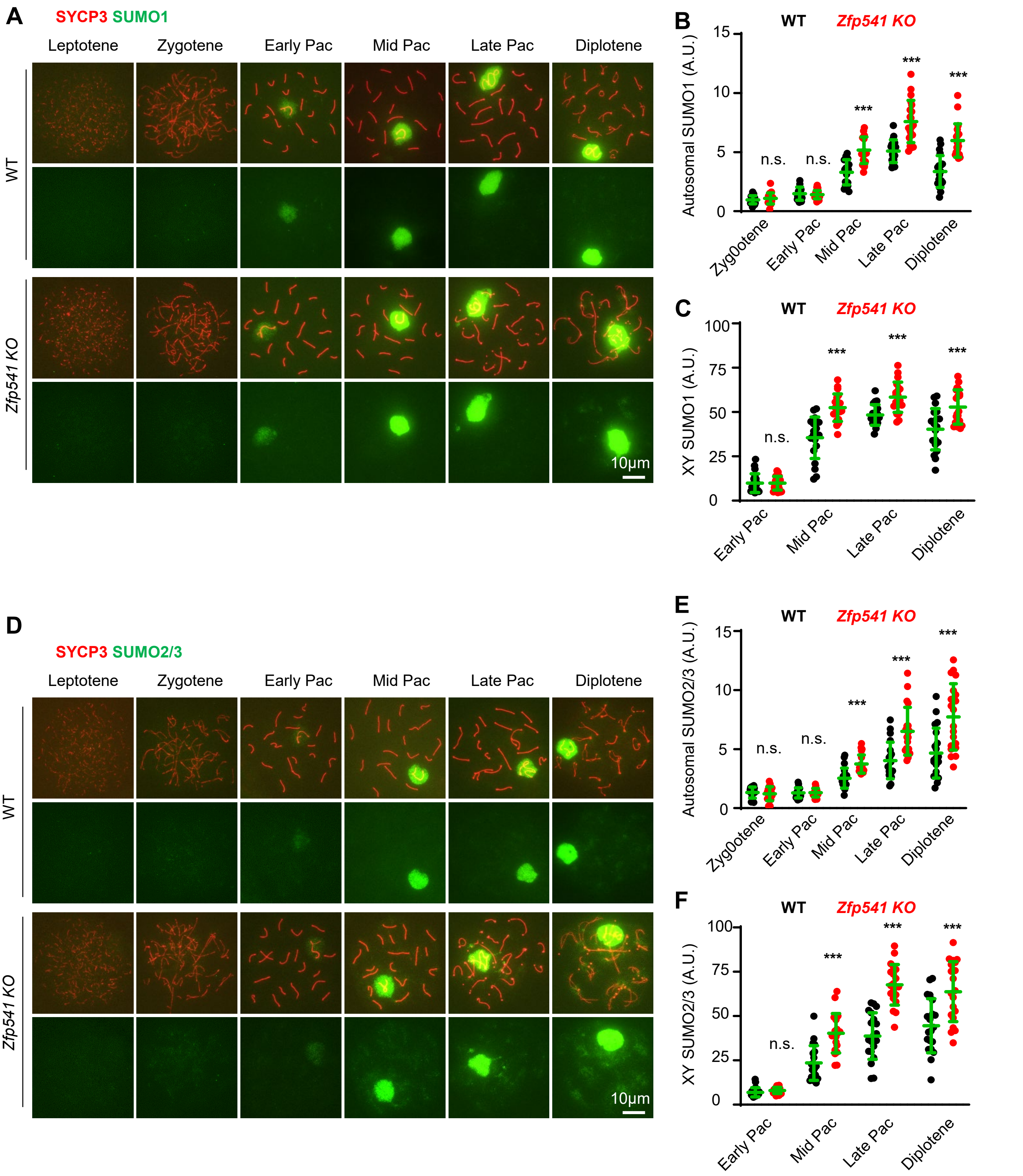


**Figure S12. *Zfp541$^{-/-}$ Kctd19$^{-/-}$* spermatocytes show increased SUMO signal.**

**(A-C)** Representative images (A) and quantification (B, C) to show SUMO1 signal on autosomal and XY chromosomes, respectively. n=20 nuclei for each genotype at each stage; Error bar, mean ± SD; n.s. (not significant), $p \geqslant 0.05$; ***, $p<0.001$; two-tailed student's t-test. **(D-F)** Representative images (D) and quantification to show SUMO2/3 signal autosomal and XY chromosomes, respectively. n=20 nuclei for each genotype at each stage; Error bar, mean ± SD; n.s. (not significant), $p \geqslant 0.05$; ***, $p<0.001$; two-tailed student's t-test.

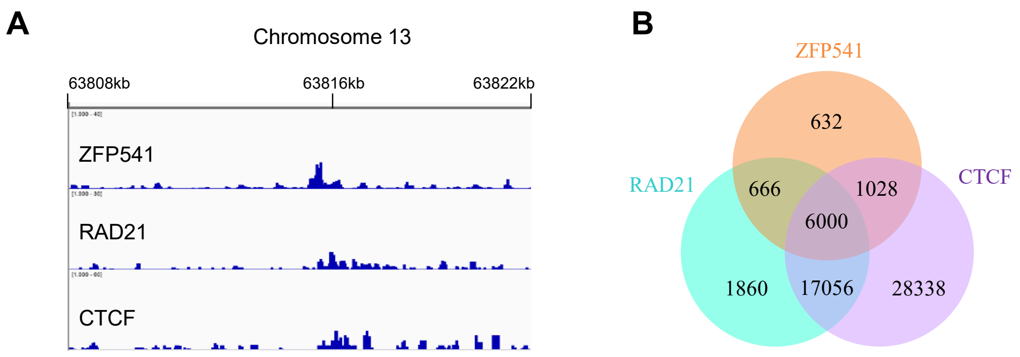


**Figure S13. Colocalization for ZFP541, CTCF, and RAD21.**

**(A)** Genome browser snapshot to show the localization for ZFP541, CTCF, and RAD21. **(B)** Venn plot to show the numbers of overlapped binding peaks between ZFP541, CTCF, and RAD21.
